# Seasonal Flooding Disrupts Expected Depth‐Dependent Patterns of Mineral Associated Carbon, Nitrogen, and Phosphorus Across Land Uses

**DOI:** 10.1111/gcb.70860

**Published:** 2026-04-17

**Authors:** Hannah P. Lieberman, Christian von Sperber, Rachael Harman‐Denhoed, A. Stuart Grandy, Cynthia M. Kallenbach

**Affiliations:** ^1^ Department of Natural Resource Sciences Macdonald Campus of McGill University Sainte‐Anne‐de‐Bellevue Québec Canada; ^2^ Department of Geography McGill University Montréal Québec Canada; ^3^ Department of Natural Resources and the Environment University of New Hampshire Durham New Hampshire USA; ^4^ Faculty of Land and Food Systems University of British Columbia Vancouver British Columbia Canada

**Keywords:** carbon, land use, mineral associated organic matter, nitrogen, organic matter persistence, phosphorus, seasonal flooding, subsoil

## Abstract

Approximately 25%–70% of soil organic matter (OM) is stored below 30 cm, rendering subsoil OM cycling an important control on OM persistence. With climate change, soils are experiencing new pedoclimatic disturbances like seasonal flooding that can destabilize mineral associated organic matter (MAOM) and undermine carbon (C), nitrogen (N), and phosphorus (P) persistence. Our current understanding of how OM cycles down the soil profile is largely based on well‐drained soils. Thus, it remains uncertain if expected depth‐dependent trends and subsoil OM persistence differ in soils subjected to seasonal flooding across land uses. We collected 1‐m‐deep soil cores within a seasonal floodplain, representing a land‐use gradient: forest, pasture, and cropland. We compared MAOM concentrations (C, N, and P), stoichiometry, isotopic, and molecular chemical composition, in combination with microbial biomass and carbon use efficiency at four soil depths to understand the transformation and persistence of OM with depth. We found that the composition of OM by depth in our soils diverges from common trends in well‐drained soils. For example, in well‐drained soils, we expect a decrease in mineral associated C:N, and an increase in polar compounds with depth, as these compounds are thought to be more persistent. Instead, we found an increase in MAOM C:N in the forest and cropland, and an increase in non‐polar C rich compounds in all land uses with depth. The pasture varied from the forest and cropland, with OM composition largely unchanged with depth. Our results suggest that the extensive root system in the pasture causes desorption of older compounds resulting in younger, plant derived compounds at all depths. We propose that the controls on persistence differ in a seasonal floodplain, where MAOM persistence is shaped more by compound resistance to hydrological stress than by formation pathways.

## Graphical abstract

The subsoil (< 30 cm) stores ~25%–70% of soil organic matter (OM), rendering it critical for carbon (C), nitrogen (N), and phosphorus (P) persistence. Our understanding of how OM cycles down the soil profile largely comes from well‐drained soils. Yet seasonal flooding can destabilize mineral‐associated organic matter (MAOM), the most persistent OM pool, and may shift MAOM depth dependent trends. We collected 1‐m‐deep soil cores across a forest, pasture, and cropland in a seasonal floodplain and analyzed MAOM concentrations (C, N, P), stoichiometry, isotopic composition, molecular composition in combination with microbial biomass, and carbon use efficiency at four depths. We found that in these seasonally flooded soils, subsoil OM composition diverged from patterns in well‐drained soils‐ compounds with characteristics that typically increase persistence in well drained soils (i.e., polar, nutrient‐rich) were more prone to loss, whereas C rich, insoluble, non‐polar compounds accumulated at depth. We further observed that land‐use effects diminished with depth, except in pasture, where we hypothesize that compounds relatively resistant to flooding may be displaced by deep root inputs, leading to faster‐cycling and younger subsoil MAOM. These results indicate that in flood‐prone soils, MAOM persistence is controlled more by resistance to hydrological stress than by formation pathways.

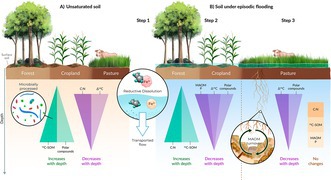

## 1 Introduction

Organic matter (OM) in the subsoil is a significant carbon (C) sink, storing an estimated 25%–70% of total soil C below 30 cm (Balesdent et al. 2018; Harrison et al. 2011; Jobbágy and Jackson 2000). Subsoil OM is considerably more persistent than topsoil OM, especially the mineral associated organic matter (MAOM) pool (Georgiou et al. 2022). This persistence is attributed to enhanced aggregate and mineral protection as well as greater physical separation between microbes and OM due to increased bulk density and reduced microbial biomass, O_2_, and OM at depth (Hicks Pries et al. 2023 and references within). Yet, subsoil MAOM is also increasingly recognized as sensitive to destabilization under climatic shifts, like changes in moisture regimes (Hicks Pries et al. 2017; Mikutta et al. 2019; Ofiti et al. 2021; Soong et al. 2021). How subsoil MAOM responds to environmental change is complex, as the extent of MAOM destabilization likely depends on the type of disturbance, the initial OM accumulation mechanisms, and the microbial community's capacity to metabolize OM. In many environments, OM composition follows a predictable depth trend that can inform how OM accumulates and persists at depth, and its vulnerability to environmental change (Hicks Pries et al. 2023; Kaiser and Kalbitz 2012; Rumpel and Kögel‐Knabner 2010). However, in soils with frequent seasonal flooding, typical depth‐driven MAOM trends may be eclipsed, shifting how subsoil MAOM accumulates and persists.

OM typically enters subsoil MAOM through root inputs or via transport as soluble OM in pore water, undergoing multiple transformations by microbial processing and selective sorption/desorption (Leinemann et al. 2018; McDowell and Likens 1988; Mikutta et al. 2019). Within the MAOM pool, OM binds to mineral surfaces or pre‐existing OM, where bond strengths depend on mineral surface properties and OM composition (Kleber et al. 2021). MAOM chemical composition directly impacts its long‐term persistence, as different bonds vary in sensitivity to environmental changes and destabilization (Bailey et al. 2019; Kleber et al. 2021).

MAOM composition and its depth trends can be influenced by land use (Jobbágy and Jackson 2000). Land use shapes OM inputs and its movement through the soil profile primarily by influencing belowground inputs, microbial processing, and preferential flow paths (Sulman et al. 2020). Changes in topsoil OM inputs and microbial activity can reverberate through the soil profile, shifting depth‐driven trends of OM persistence (Lange et al. 2023; Xu et al. 2021). For instance, low C:N litter inputs might accelerate surface decomposition and increase microbial carbon use efficiency (CUE) (Manzoni et al. 2012), affecting the quantity and composition of compounds reaching the subsoil. In temperate forest ecosystems, soluble OM transported from surface horizons is a dominant subsoil input, often having already undergone multiple cycles of microbial decomposition before it is deposited at depth (Jobbágy and Jackson 2000; Souza et al. 2023). In contrast, grasslands, like pastures, have deep extensive fine root systems that contribute more belowground plant inputs to subsoil MAOM than forests and croplands (Bai and Cotrufo 2022). Cropland systems shift OM distribution through tillage (Luo et al. 2010), and fertilizer and manure applications increase soluble N‐ and P‐rich OM that can leach to the subsoil (Angers et al. 2010; Samson et al. 2021). These differences in OM sources and transport pathways can lead to distinct MAOM compositions across land uses (Sokol et al. 2022), impacting its susceptibility to disturbances.

Despite these possible ecosystem‐driven differences, certain depth‐dependent trends emerge across land uses. For instance, microbial‐derived and polar compounds, typically N and P rich, are thought to accumulate within the subsurface MAOM pool, increasing with depth (Hicks Pries et al. 2023; Kaiser and Kalbitz 2012; Rumpel et al. 2012). Depth‐dependent isotope patterns also develop where δ^13^C often increases and Δ^14^C decreases with depth. This δ^13^C depth trend mirrors the narrowing of C:N and C:P, as decomposed compounds approach the stoichiometry of microbial biomass (Liebmann et al. 2020). The C age also often increases with depth as evidenced by a decrease in Δ^14^C, reflecting possible slow turnover time of subsoil C and sometimes contributions of pedogenic C from parent material (Shi et al. 2020; Trumbore et al. 1992). Thus, conceptual models (e.g., Hicks Pries et al. 2023; Kaiser and Kalbitz 2012; Rumpel and Kögel‐Knabner 2010) and numerous field studies (e.g., Guggenberger and Zech 1994; Kaiser et al. 2004; Lange et al. 2023; Leinemann et al. 2018; Liebmann et al. 2020; Pei et al. 2025; Sanderman et al. 2008) suggest that plant‐derived compounds, microbial biomass, CUE, and MAOM C:N and C:P generally decrease with depth, while microbial‐derived compounds, polar compounds, δ^13^C, and OM age relatively increase.

Yet, these expected trends and MAOM persistence mechanisms at depth are largely based on upland, well‐drained soils (Balesdent et al. 2018; Kaiser and Kalbitz 2012; Rumpel et al. 2012; von Fromm et al. 2024). Consequently, it is unknown if similar depth‐dependent trends occur in periodically flooded soils, like those in a seasonal floodplain. With climate change, short‐term flooding is increasing globally (Hirabayashi et al. 2021), exposing more soils to periods of water saturation. Flooding could diminish (or reverse) these depth‐dependent trends by shifting destabilization dynamics or OM composition. First, flooding may enhance MAOM destabilization and its subsequent loss via leaching or microbial respiration through: (1) anoxic and reducing conditions causing reductive dissolution of iron (Fe)‐oxides that releases sorbed OM (Lin et al. 2018; Yang et al. 2025); (2) aggregate breakdown and the release of occluded OM into the soluble bioavailable pool (Fierer and Schimel 2003), especially in systems that experience freeze–thaw cycles which amplify changes to aggregates and pore structure (Bailey et al. 2019); and (3) increased pore connectivity that enhances microbial access to previously bio‐inaccessible OM (Lehmann et al. 2020; Patel et al. 2021). Second, flooding impacts OM transport, sorption/desorption processes, and microbial function, including CUE, likely altering MAOM composition at depth (Heckman et al. 2023; Tipping et al. 2012; von Fromm et al. 2025). Increased and rapid downward transport of soluble OM may bypass sorption sites at shallower depths (Fröberg et al. 2007), and reduced microbial OM decomposition in the topsoil under water saturation could lead to more plant‐derived OM transported to lower depths. Alternatively, higher OM transport rates may increase microbial biomass and access to OM at lower depths (Schimel et al. 2011; Xiang et al. 2008), possibly enhancing subsoil CUE and decomposition compared to their well‐drained counterparts. Thus, we might expect that flood‐driven alterations to soluble OM entering subsoil MAOM cause distinct differences in the quantity and composition of subsoil MAOM compared to their well‐drained counterparts.

Notably, unlike more permanently flooded soils, seasonal floodplains and poorly drained soils fluctuate between periods of anaerobic and aerobic conditions. These wet‐dry cycles can destabilize MAOM, with impacts showing a hysteresis (Haines 1930; Patel et al. 2021; Xiang et al. 2008). Seasonally flooded soils can also experience high levels of OM leaching and runoff during flood retreat (Bridge 2009). Thus, the impact of short‐term flooding on subsoil MAOM may also differ from observed trends in wetlands and rice paddies which are saturated for longer periods of time.

Our objective was to understand how seasonal flooding impacts depth‐dependent changes in soluble OM and MAOM composition across land uses (forest, pasture, and cropland). In combination with microbial biomass and carbon CUE, we characterized MAOM concentrations (C, N, and P), stoichiometry, isotopic, and molecular chemical composition through 1‐m soil profiles to: (1) determine if MAOM in seasonally flooded soils deviates from expected depth trends commonly observed in well‐drained soils; (2) if so, how land use modulates these divergences; and (3) use our findings to develop a greater understanding on the controls of OM persistence in floodplain soils. We expected to find land use driven differences in MAOM composition especially near the surface. These land use driven changes in MAOM would lead to distinct responses to flooding, reflected in seasonal differences in subsoil MAOM concentration, composition, and persistence among the three land uses.

## 2 Methods

### 2.1 Site Description

We conducted our study using soils collected from Baie‐du‐Febvre, Quebec, Canada, a township within the Lake Saint Pierre (LSP) floodplain in southeastern Quebec, Canada (Figure S1a). The floodplain is 28,000 ha and includes the largest freshwater wetland along the Saint Lawrence River (Hudon et al. 2018). Baie‐du‐Febvre has a mean annual temperature of 5.6°C, a mean annual precipitation of 947.4 mm, and a mean annual snowfall of 212.9 cm (Government of Canada Climate Normal from weather stations within 20 km of site locations). The LSP floodplain, including our sites, experiences near‐annual springtime flooding due to snowmelt, typically beginning in April and lasting between 5 and 9 weeks (Figure S1c). Occasional shorter summer flooding also occurs from precipitation due to the poorly drained nature of these soils (Jean and Létourneau 2011). In the sampling year, the flood period was unusually low, flooding for less than a week (Campeau et al. 2024). The soils at our sites are gleysols developed over an ancient sandy river terrace and marine sediment derived from the postglacial Champlain Sea, overlaid by alluvial deposits (Quebec Ministry of Natural Resources and Forests, and Soils of Canada (Landscape of Canada database)).

The natural ecosystems of the region are Maple (*Acer* spp.) dominated forests and wetlands. Historically, managed LSP ecosystems were dominated by perennial forage crops and pasture; however, beginning in 1950s, these land uses were largely converted to annual cropland, predominantly corn ( *Z. mays* ) and soybean ( *G. max* ) production (Dauphin and Jobin 2016; Jobin and Brodeur 2023). We sampled from three sites representative of this land use conversion: forest (46°10′N, 72°41′W), pasture (46°11′N, 72°39′W), and cropland under a corn and soybean rotation (46°09′N, 72°42′W) (Figure S1b). The forest site is an unmanaged deciduous silver maple‐dominated (*Acer saccharinum L*.) forest with a 50.5% clay topsoil and topsoil pH of 8.2. To the best of our knowledge this site is remnant forest, with no history of land conversion or management (Jobin and Brodeur 2023). The pasture has been planted in perennial grasses dominated by Timothy grass (*p. pratense*), Couch grass ( *A. repens* ) and Rattlesnake Manna grass ( *G. canadensis* ) since at least 2012 but likely longer, with a 32.5% clay topsoil and a topsoil pH of 7.7. This site is mowed 1–2 times a year. There is no evidence that this site was previously used for annual row crops. The cropland site is under a corn‐soybean rotation that undergoes tillage and conventional fertilizer inputs and is left bare in the winter with 42.7% clay topsoil and a topsoil pH of 7.5.

### 2.2 Sampling Design

We collected 1‐m‐deep, 4‐cm diameter soil cores (*n* = 4) from each land use site twice, in May 2021 (spring) approximately 2 weeks after flood retreat and October 2021 (fall) (Figure S1c,d). These sampling times were chosen to capture conditions that were either: (1) recently flooded (spring) or (2) drier and after the growing season following large litter inputs from post‐harvest crop residues or pasture and deciduous forests senesces (fall). Within each site, a rectangle 4.3 × 8.3 m was selected on the side closest to the lakeshore and we randomly sampled 4 cores within each rectangle. For the fall sampling, cores were taken at the same GPS coordinate as the spring cores to ensure similarity in soil pedological variables between sampling times. During the spring sampling, the cropland had corn residue remaining from the previous summer's planting while in the fall, soybean was recently harvested. Plant cover, both living and ground litter, was collected directly above each core and oven dried (60°C) after sampling. Cores were subdivided in the field into 4 sections: 0–10, 30–40, 60–70 and 85–100 cm. The 85–100 cm sections had gleying features, distinct from the rest of the soil core. Samples were kept frozen (−20°C) until further processing. Soil moisture content (SMC) was determined at the time of processing based on the mass difference between field‐moist and oven‐dried (105°C) soil.

### 2.3 Mineral Associated C, N, P, and Metal Concentrations

We isolated the mineral associated fraction using a size fractionation technique (Jilling et al. 2020; Tiessen and Stewart 1983). Specifically, 10 g dry soil was shaken in 50 mL deionized water for 18 h on an end‐to‐end shaker at 280 osc/min; we then removed the particulate organic matter by flotation and sonicated the samples at 80% amplitude for 4 min and 36 s to reach 440 joules/ml (Branson SFX250 Sonifier, Brookfield, CT, USA). Following sonication, the clay and fine silt fractions (< 53 μm) were isolated by rinsing the dispersed soil with deionized water over a 53‐μm sieve. This fraction was subsequently dried at 105°C for 48 h and ground by mortar and pestle.

MAOM C and N were quantified using a flash combustion elemental analyzer (Costech EA ECS 4010, Valencia CA, USA). Inorganic C in our soil was considered a negligible fraction as determined by the fizz test (Baldock et al. 2013). Samples were sent to the Natural Resources Analytical Laboratory (NRAL) at the University of Alberta for MAOM P, Fe, aluminum (Al), and calcium (Ca) analysis, where samples were first digested using a MARS microwave digestion system and concentrated nitric acid method (U.S. EPA 2007). These digestions were then analyzed on an inductively coupled plasma‐optical emission spectrometer (U.S. EPA 2014; ICP‐OES; Thermo iCAP6300 Duo (N. America), Thermo Fisher Corp., Cambridge, UK).

### 2.4 MAOM Isotopes

We measured the δ^13^C and δ^15^N of the MAOM fraction in the Ecological Tracers Lab at McGill University (Sainte‐Anne‐de‐Bellevue, QC, Canada). Natural abundance ^13^C and ^15^N were measured using a Thermo Scientific EA Isolink Flash Elemental Analyzer with oven‐ramped gas chromatography paired with a Delta V Plus Isotope Ratio Mass Spectrometer (IRMS) configured to simultaneously measure C and N isotopes. Inorganic C was removed by HCl fumigation before analysis (Harris et al. 2001). Isotopic values were calibrated according to Land‐Miller et al. (2024), for details see Methods S1.

We sent one composite sample of each land use and depth for radiocarbon (Δ^14^C) analysis at the André E. Lalonde National Facility in Accelerator Mass Spectrometry at the University of Ottawa (Ottawa, ON, Canada). MAOM Δ^14^C was quantified on an Ionplus AG MICADAS (Mini Carbon Dating System). The ^12,13,14^C + 1 ions were measured after tandem acceleration by a terminal at 185 kV and equipped with He stripping. Isotope data were processed using the BATS data reduction software as described by Wacker et al. (2010). ^14^C/^12^C ratios were background‐corrected and fractionation‐corrected to δ^13^C_PDB = −25 permil.

### 2.5 Mineral Associated Organic Matter Molecular Chemistry

We used pyrolysis‐gas chromatography/mass spectrometry (py‐GC/MS) to examine the molecular chemistry of MAOM for all spring soil samples and pulverized litter according to Kallenbach et al. (2015). Soil or litter samples were first pyrolyzed (CDS Pyroprobe 6150) for 20 s at 650°C and then transferred to a GC (Trace 1610, Thermo Scientific) via a 300°C transfer line. Compounds were separated on a fused silica GC column (60 m × 0.25 mm) heated from 40°C to 270°C (5°C min−1) with a final hold at 310°C for 20 min. Compounds were then immediately transferred to an ISQ 7610 single quadrupole MS (Thermo Scientific) via a 300°C transfer line and ionized at 70 eV (scan range 40–650) with the source temperature held at 275°C. Compound peaks were deconvoluted with AMDIS (chemdata.nist.gov; v.2.65), and identified with the NIST compound library and published literature. We determined individual compound relative abundance based on total sample peak area and then classified compounds into classes: lipids, lignin derivatives, polysaccharides, protein, non‐protein N‐bearing, phenolics, and unknown origin. Unknown origin refers to compounds that could not be attributed to a specific class.

To further assign functional properties to each compound, we categorized the compounds into four levels of solubility based on their log K_ow_ coefficient (Chen et al. 2020), defined as the ratio between a compound's concentration in n‐octanol and its concentration in water. A log K_ow_ < 1 indicates a compound is more soluble in water and > 1 it is more soluble in non‐polar octanol. Thus, the log K_ow_ also provides an indication of polarity, where a lower value correlates with a higher degree of polarity and solubility. We determined the log K_ow_ of each compound from pubchem (Kim et al. 2025) and defined the levels of solubility as: soluble (< 1), slightly insoluble (1–2), insoluble (2–5), and highly insoluble (> 5). The value of 5 was selected as it is the log K_ow_ cutoff used by the United Nations, where compounds are considered highly hydrophobic and bio‐accumulative (Annex 2008; Gimeno et al. 2024).

### 2.6 Water Extracted C, N, and P

We measured water extractable organic C (WEOC), total N (WEN), and inorganic P (WEP) by adding 40 mL of deionized water to 10 g (fresh weight) slowly thawed soil, followed by shaking for 30 min on an end‐to‐end shaker, centrifuging for 20 min at 3600 *g* and then filtering to 2.5 μm. Here we operationally define the water extracted C, N, and P pools at < 2.5 μm as our soluble pools. While this size fraction includes non‐soluble and undissolved compounds, it better captures all OM that might be mobile in water, and thus more vulnerable to loss, biological uptake, and transformation with flooding. We measured WEOC and WEN concentrations on a TOC/N analyzer (Shimadzu Corp, Kyoto, Japan). WEP was measured on a 96‐well plate spectrometer (BioTek Instruments, Winooski, Vt, USA) at 630 nm using malachite green (Ohno and Zibilske 1991).

### 2.7 Microbial Biomass and Carbon Use Efficiency

Salt‐extractable microbial biomass C (MBC) and N (MBN) were analyzed using a modified chloroform fumigation extraction method (Wu et al. 1990). We fumigated 8 g of slowly thawed soil with chloroform for 24 h and then extracted these soils with 40 mL of 0.5 M K_2_SO_4_. A corresponding set of samples was extracted in the same manner but not fumigated. Fumigated and unfumigated extracts were analyzed on a TOC/N analyzer (Shimazdu Corp, Kyoto, Japan). MBC and MBN were calculated as the difference between the fumigated and unfumigated C and N concentrations. As *Kec* (extraction efficiency) values likely vary by depth, we report salt‐extractable MBC and MBN without further adjustments (Dictor et al. 1998; Tessier et al. 1998).

Microbial CUE was determined for the 0–10 and 85–100 cm depths for each site and season. We measured CUE using a ^13^C glutamic acid label and compared the amount of substrate incorporated into microbial biomass to the total uptake amount of ^13^C (^13^CO_2_‐C plus MB^13^C) following Kallenbach et al. (2015). For details see Methods S2.

### 2.8 Data Analyses

Statistical analyses were performed in R v 4.4.1. For all statistical analyses, significance was defined as *p* < 0.05. Within this dataset, there are post hoc pairwise comparisons among land uses, depths, and seasons that are not ecologically relevant to our study questions. Hence, we were interested in understanding only the post hoc pairwise comparisons: (1) by depth within a single land use, (2) within a single depth among land uses, or (3) between seasons within a single depth and land use.

First, we looked at the effects of land use, depth, and season and their interactions using a three‐way ANOVA followed by a Tukey post hoc test (Table S1). For all variables except soil moisture content (SMC), and MAOM C:N, we found no effect of season that included post hoc comparisons of interest described above. For MAOM C:N only pasture at 60–70 cm differed by season (*p* = 0.01). Thus, we combined the spring and fall samples within one site and depth for greater statistical power for every variable except SMC. We then determined differences in depth, land use, and their interaction within an individual variable using a two‐way ANOVA followed by a Tukey post hoc test. Normality and homoscedasticity of residuals were evaluated with diagnostic plots and Shapiro–Wilk tests. Data were log transformed, and outliers, determined by diagnostic and qq‐plots, were removed as necessary. We used a generalized linear model with a Gaussian distribution to compare differences in δ^13^C as this was non‐normally distributed under any transformation.

We used a non‐metric dimensional scaling ordination (NMDS) to determine land use and depth differences in overall MAOM molecular chemical compound composition using a Bray–Curtis dissimilarity matrix. The effects of depth and land use and their interaction on MAOM compound composition were each evaluated using a Permanova. We used an ANOVA to evaluate the effects of depth and land use on the relative abundance of each compound K_ow_ level and each class. Data were square root transformed as necessary for normality.

## 3 Results

### 3.1 Soil Moisture Content

The SMC was affected by season (Table S1), but only in the forest, with a higher moisture content in the spring than the fall for every depth (Table S2; *p* < 0.001). The three land uses differed in depth‐driven moisture patterns (Tables S1 and S2), where the forest was wetter near the surface, but the cropland and pasture were wettest deeper in the soil. The forest spring SMC decreased by 65% from 0–10 cm (101.8%; 97% estimated water filled pore space (WFPS)) to 30–40 cm (35.4%; 60% estimated WFPS) and remained at this lower SMC in the two lowest depths. The fall forest soil showed a similar pattern of decreasing SMC with depth. In contrast, pasture SMC increased with depth in the fall and spring, from a mean 33.5% (49% estimated WFPS) at 0–10 cm to a mean 47.75% (98% estimated WFPS) at 85–100 cm (*p* < 0.005). The cropland SMC also increased from 23.6% (37% estimated WFPS) in the topsoil to a mean 36.9% (71% estimated WFPS) at the 60–70 cm depth (*p* < 0.005).

We observed differences in SMC among all the land uses, but this was confined to the 0–10 cm and 85–100 cm depths (Table S2). At 0–10 cm in the spring, forest had the highest SMC, followed by pasture and cropland (*p* < 0.01). In the fall, forest and pasture had a higher SMC than cropland. Whereas, in the 85–100 cm depth, SMC only differed in the fall, where pasture (48.5% SMC) and cropland (40.3% SMC) had higher SMC than forest (32.7% SMC) (*p* < 0.05).

### 3.2 Mineral Associated C, N, and P

Soil depth, land use, and their interactions all had significant effects on MAOM C, N, P, and MAOM stoichiometry (Table S1; *p* < 0.05). Seasonal effects were limited to MAOM C:N within the pasture. Based on these significant main effect differences, below we report post hoc pairwise comparisons for depth‐driven changes within a land use, and comparisons among land uses within a given depth.

#### 3.2.1 MAOM C

**FIGURE 1:**
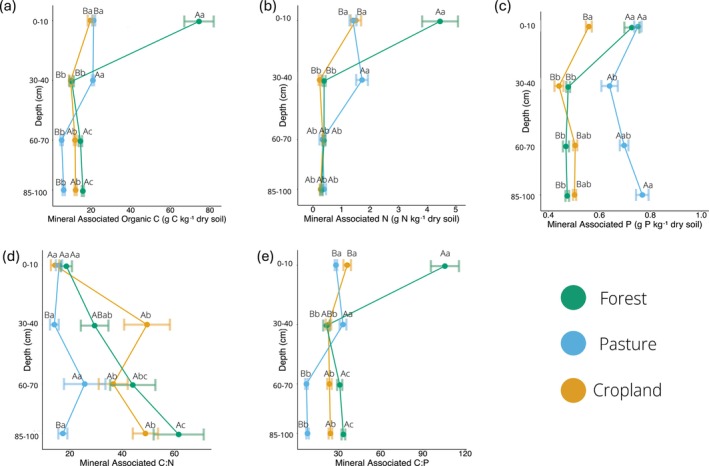
Mineral associated organic C (a), mineral associated N (b), mineral associated P (c), mineral associated C:N (d) and mineral associated C:P (e) concentrations (g kg^−1^ dry soil) for forest, pasture, and cropland sites at 0–10, 30–40, 60–70 and 85–100 cm depths with seasons combined. Points represent the mean and error bars represent standard error. X‐axis scales differ among panels. Different upper‐case letters indicate significant pairwise differences within one depth among land uses (*p* < 0.05); *n* = 8, *d.f. =* 2. Different lower‐case letters indicate significant pairwise differences within one land use among depths (*p* < 0.05); *n* = 8, *d.f. =* 3.

MAOM C content decreased with depth across all land uses (Figure 1a). Forest MAOM C decreased most dramatically from 0–10 cm (74.5 g kg^−1^) to 30–40 cm (10.3 g kg^−1^), decreasing 86% (*p* < 0.001). At 60–70 cm, forest MAOM C trended towards an increase (14.7 g kg^−1^) compared to the 30–40 cm depth (*p* = 0.07); but it only significantly increased again at 85–100 cm (15.9 g kg^−1^; *p* < 0.01). MAOM C content also decreased in the pasture with depth. However, this decrease occurred at the 60–70 cm depth (5.2 g kg^−1^), where it decreased by 76.2% from 30–40 cm (21.0 g kg^−1^; *p* < 0.001) and was similar to MAOM C at the 85–100 cm depth. The cropland showed a similar pattern to the forest, with a 50% decrease in MAOM C at the same 30–40 cm depth (*p* < 0.001), from 19.7 g kg^−1^ to 9.8 g kg^−1^, which endured at the two lower depths.

At 0–10 cm forest MAOM C was 3.8 and 3.5 times greater than the cropland and pasture respectively (*p* < 0.001). However, by the 30–40 cm depth, the difference between forest and cropland MAOM C content disappeared (*p* > 0.05). At the 30–40 cm depth, the pasture contained higher MAOM C content (21.0 g kg^−1^; *p* < 0.001) than both forest and cropland. In contrast below 60 cm, pasture MAOM C concentration was lower than forest and cropland (*p* < 0.001).

#### 3.2.2 MAOM N

MAOM N content (Figure 1b) largely followed the same pattern as MAOM C, decreasing with depth in all three land uses. MAOM N content, especially below 30 cm, was low in all land uses and depths (< 2 g kg^−1^). Forest MAOM N content decreased by 91% between 0–10 (4.45 g kg^−1^) and 30–40 cm (0.39 g kg^−1^; *p* < 0.001) and did not change at lower depths. Pasture MAOM N content showed the same pattern as MAOM C, where an 80.5% decrease occurred from 30–40 to the 60–70 cm depth (0.33 g kg^−1^; *p* < 0.001), which remained at the 85–100 cm depth. Cropland MAOM N content also behaved similarly to its MAOM C, with a decrease from 0–10 (1.50 g kg^−1^) to 30–40 cm (0.24 g kg^−1^; *p* < 0.001), with no changes observed below this depth.

Forest MAOM N content at 0–10 cm (4.45 g kg^−1^) was 3.2 and 2.9 times greater than pasture (1.4 g kg^−1^; *p* < 0.001) and cropland (1.50 g kg^−1^; *p* = 0.001), respectively. In contrast, at 30–40 cm, pasture MAOM N content was greater than forest and cropland (*p* < 0.001). MAOM N content did not differ between any land‐use in the lowest two depths (*p* > 0.05).

#### 3.2.3 MAOM P

MAOM P behaved similarly to MAOM C and N in the forest, decreasing at 30–40 cm by 34%, with no further changes at lower depths (Figure 1c). There was also a decrease between 0–10 and 30–40 cm in pasture (*p* < 0.01) and cropland (p < 0.001), decreasing by 15% and 21% respectively. However, in the pasture at 60–70 cm there was a slight increase in MAOM P, increasing significantly at 85–100 (0.77 g kg^−1^) which did not differ from 0 to 10 or 60 to 70 cm (*p* > 0.05). Cropland MAOM P increased slightly at 60–70 (0.51 g kg^−1^) from 30 to 40 cm (0.44 g kg^−1^), but the two lowest depths did not differ from the top two (*p* > 0.05).

At 0–10 cm, forest (0.73 g kg^−1^) and pasture (0.75 g kg^−1^) contained higher MAOM P than cropland (0.56 g kg^−1^). However, at 30–40 cm, the forest and cropland MAOM P were similar to each other, while pasture MAOM P (0.64 g kg^−1^) was 1.4 times higher than forest and cropland (*p* < 0.001). This pattern continued in the lower two depths.

### 3.3 MAOM Stoichiometry

We found that MAOM C:N increased with depth in the forest and cropland but did not change in the pasture (Figure 1d; *p* > 0.05). There was large variability in the forest and cropland sites indicating high levels of heterogeneity. MAOM C:N increased at the 60–70 cm depth in the forest site compared to the 0–10 cm depth, from 16.9 to 46.3 (*p* = 0.01), remaining higher at 85–100 cm depth (61.6). In the cropland, MAOM C:N increased from 14.3 at 0–10 to 49.5 at 30–40 cm (*p* < 0.001), with no further changes at lower depths.

Differences in MAOM C:N among the land uses only emerged at 30–40 cm and again at the 85–100 cm depth. At 30–40 cm, cropland C:N (49.5) was higher than the pasture (14.1; *p* < 0.001). In the 85–100 cm depth, both the forest (61.7) and cropland (50.4) had higher MAOM C:N than pasture (17.3). Lastly, we observed a seasonal effect on MAOM C:N, but post hoc testing showed this was limited to the pasture at 60–70 cm which had a higher C:N in the spring than the fall (*p* = 0.01).

Unlike MAOM C:N, both MAOM C:P and N:P generally decreased from the topsoil 0–10 cm to the subsoil below 30–40 cm for forest and cropland and below 60–70 cm for pasture (Figure 1e; Table S3). At 30–40 cm, pasture had a higher MAOM C:P (33.3) than forest (21.6; *p* < 0.01). Yet, at 60–70 cm, forest (31.0; *p* < 0.001) and cropland (23.7; p < 0.001) had a MAOM C:P 3 and 4 times greater than pasture (7.4) respectively, which remained at 85–100 cm.

### 3.4 Mineral Associated Fe, Ca, and Al

**FIGURE 2:**
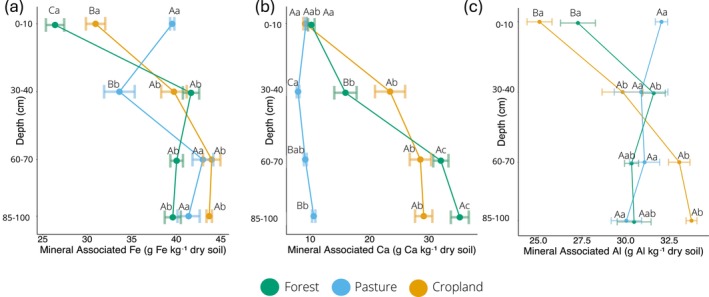
Mineral associated Fe (a), mineral associated Ca (b), and mineral associated Al (c) concentrations (g kg^−1^ dry soil) for forest, pasture, and cropland sites at 0–10, 30–40, 60–70 and 85–100 cm depths with seasons combined. Points represent the mean and error bars represent standard error. X‐axis scales differ among panels. Different upper‐case letters indicate significant pairwise differences within one depth among land uses (*p* < 0.05); *n* = 8, *d.f. =* 2. Different lower‐case letters indicate significant pairwise differences within one land use among depths (*p* < 0.05); *n* = 8, *d.f. =* 3.

We found that both depth and land use affected MAOM Fe and Ca (Table S1; Figure 2). MAOM Fe increased with depth for forest and cropland at 30–40 cm (Figure 2a; *p* < 0.001). Whereas in the pasture it decreased at 30–40 cm and then increased back to topsoil concentrations at 60–70 cm. The largest change occurred in the forest at 30–40 cm, increasing from 26.4 at 0–10 cm to 41.7 g kg^−1^ (*p* < 0.001). In general, forest had the lowest Fe content except for the 30–40 cm depth, where pasture Fe content (33.6 g kg^−1^) was the lowest. MAOM Ca content in the forest and cropland increased in the subsoil from the topsoil (Figure 2b). However, pasture Ca content largely did not change with depth and was also overall the lowest subsoil MAOM Ca concentrations. MAOM Al was only affected by depth and the interaction of land use and depth (Table S1; Figure 2c). Differences among land uses Al were limited to the topsoil with pasture containing higher MAOM Al than forest and cropland (*p* < 0.001).

### 3.5 Mineral Associated Organic δ^13^C and Δ^14^C

**FIGURE 3:**
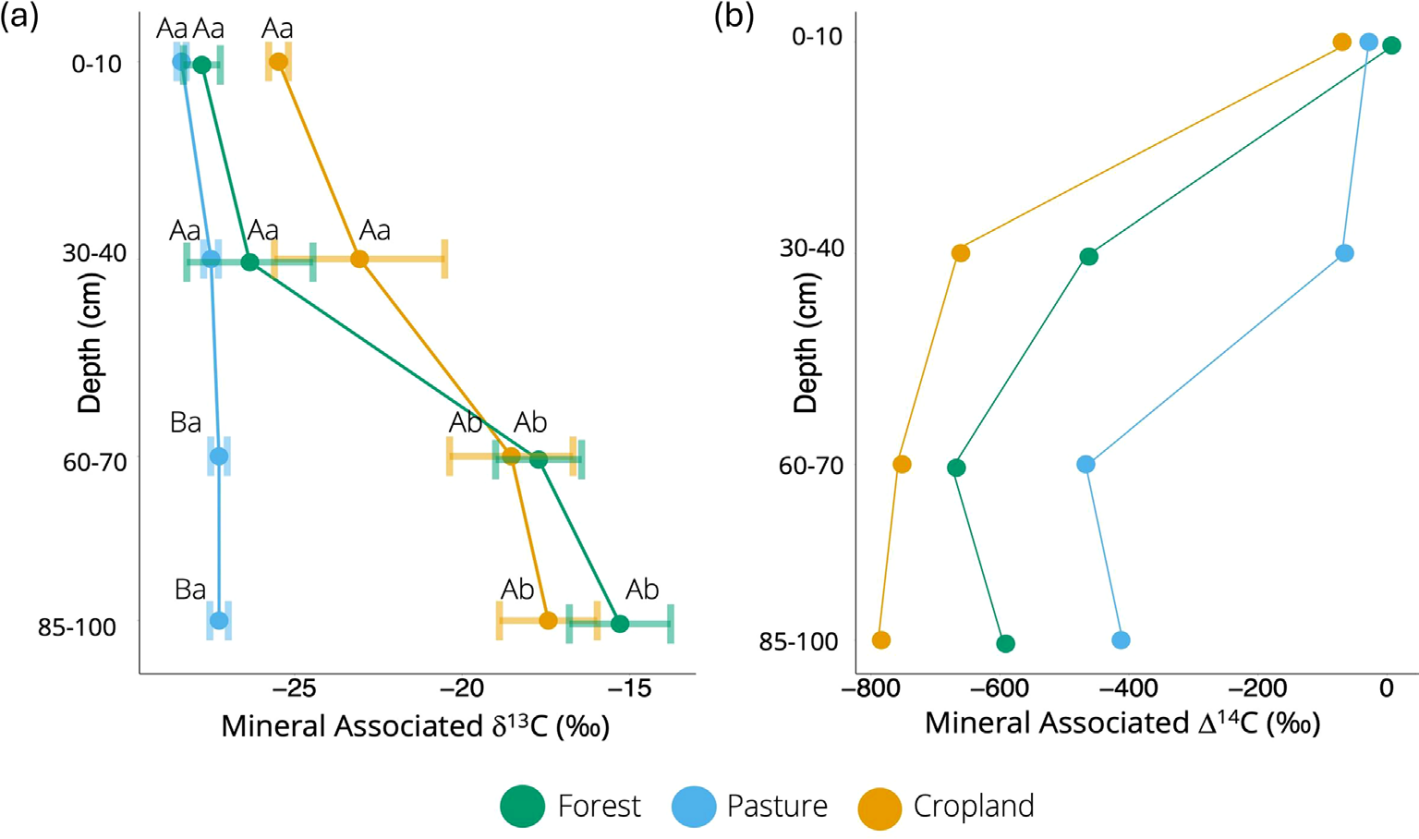
Natural abundance isotope ratio (‰) of mineral associated δ^13^C for forest, pasture, and cropland sites at 0–10, 30–40, 60–70 and 85–100 cm depths with seasons combined (a). Points represent the mean and error bars represent standard error. Different upper‐case letters indicate significant pairwise differences within one depth between land uses (*p* < 0.05); *n* = 8, *d.f. =* 2. Different lower‐case letters indicate significant pairwise differences within one land use between depths (*p* < 0.05); *n* = 8, *d.f. =* 3. Natural abundance isotope ratio (‰) of mineral associated Δ^14^C for forest, pasture, and cropland sites at 0–10, 30–40, 60–70 and 85–100 cm depths (b). Each point is one composite sample of the spring samples. *X*‐axis scales differ between panels.

We observed a depth dependent land use effect on MAOM δ^13^C, with high variability in the forest and cropland below the topsoil (Table S1; Figure 3a). MAOM δ^13^C increased with depth in the forest and cropland, but pasture MAOM δ^13^C did not change at any depth (*p* > 0.05). The increase in forest and cropland MAOM ^13^C enrichment started at the 60–70 cm depth, remaining similar at 85–100 cm. In the forest, δ^13^C was −27.7 at 0–10, increasing to −15.3 at 85–100 cm (*p* < 0.001). Similarly, the cropland increased from −25.5 at 0–10 to −17.4 at 85–100 cm (*p* < 0.001). The cropland was more enriched in MAOM ^13^C than pasture at 0–10 cm (Figure 3a), but in the lowest two depths, the forest and cropland (−17.7, and −18.5, respectively) appeared more enriched in ^13^C compared to pasture (−27.3).

As we did not have replicates for Δ^14^C, we could not run statistical analyses on these data. However, we observed different trends in the Δ^14^C signature among land uses (Figure 3b). While Δ^14^C decreased with depth in every land use, an indication of increasing age, this decrease occurred at 30–40 cm for forest and cropland but deeper, at 60–70 cm, in the pasture. We also found that pasture had the youngest MAOM C at every depth except 0–10 cm where the land uses were similar.

### 3.6 Mineral Associated Molecular Composition

**FIGURE 4:**
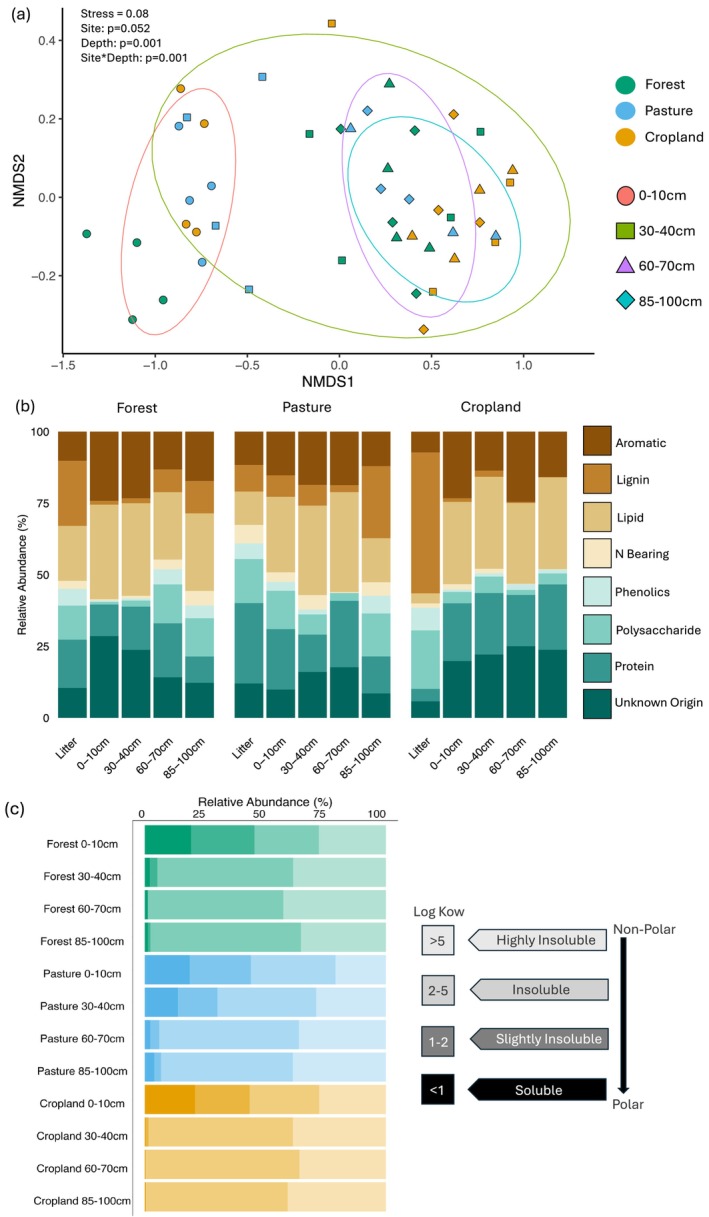
Non‐metric MultiDimensional Scaling (NMDS) ordination based on Bray‐Curtis distances of MAOM individual compound relative abundances for forest, pasture, and cropland sites at 0–10, 30–40, 60–70 and 85–100 cm depths for spring samples (a). Colors represent land use, shapes represent soil depth and ellipses represent 80% confidence intervals around group centroids by soil depth. Permanova land use*depth *p* = 0.001 *n* = 4, *d.f. =* 6; land use *p* > 0.05 *n* = 4, *d.f. =* 2; and depth *p* = 0.001 *n* = 4, *d.f. =* 3. Mean relative abundances (%) of compound classes for plant litter and mineral associated compounds (b). Mean relative abundance (%) of mineral associated compounds categorized into four levels of solubility based on their log K_ow_ (c). The log K_ow_ for compounds ranged from 15.3 to −1.0. Colors represent land use and transparency levels represent solubility levels.

We found that MAOM molecular compound chemistry was primarily separated by depth and by its interaction with land use (Figure 4a; *p* = 0.001), while land use approached significance (*p* = 0.052). In ordination space, topsoil (0–10 cm) was distinct from the other depths and topsoil forest was further separated from topsoil cropland and pasture. The lower depths across land uses were nearly indistinguishable from each other in MAOM composition ordination space.

When comparing MAOM chemistry by compound class, we observed both land use and depth effects depending on the class (Table S4; *p* < 0.05). MAOM was primarily composed of lipids, aromatics, and compounds of unknown origin (Figure 4b). Changes in the relative abundance of compound classes were predominantly driven by depth, where topsoil and/or litter generally differed from the lowest three depths (Table S4). Pasture generally contained higher N‐bearing (*p* < 0.001) and polysaccharide (*p* < 0.001) relative abundances than forest and cropland. Whereas cropland had higher compound relative abundances of unknown origin (*p* < 0.05) and lower lignin‐derived compounds (*p* < 0.01), especially at depth. Notably, pasture at 85–100 cm contained the highest lignin relative abundances (25.1%) of all land uses and depths, except for litter.

Compounds were further classified by their log K_ow_, where a decrease in log K_ow_ corresponds to increased water solubility and polarity. Compounds with a low log K_ow_ in our soils were typically polysaccharides, N‐bearing or proteins with an O, N, or P moiety (Figure 4c). In contrast, compounds with a higher log K_ow_ were more often lipids, aromatics, of unknown origin, and O/N/P free compounds. We found an increase in the relative abundance of water insoluble and non‐polar compounds (log K_ow_ > 2) with depth (Table S5; *p* < 0.001). This increase occurred at a shallower depth in the forest and cropland (Figure 4b), primarily driven by an increase in insoluble compounds (log K_ow_ 2–5). In the topsoil across all land uses, 40%–45% of MAOM compounds had a log K_ow_ < 2 (soluble and slightly insoluble), with about 20% of those compounds displaying a log K_ow_ < 1. At 30–40 cm there was a large decrease in MAOM solubility and polarity in the forest and cropland, where compounds with log K_ow_ < 2 decreased to about 1.5% in cropland and 5% in forest, which remained throughout the soil profile. In the forest and cropland sites within the three lowest depths, 58%–65% of the compounds had a log K_ow_ between 2–5, and 34%–39% of compounds were > 5. In the pasture, the decrease in MAOM solubility occurred more gradually and at a lower depth compared to forest and cropland. MAOM water soluble compounds (log K_ow_ < 1) in pasture decreased to about 14% at 30–40 cm, 2% at 60–70 cm depth, with a slight increase at 85–100 cm to about 4%.

### 3.7 Water Extracted Organic C, Total N, and Inorganic P

Soil WEOC, WEN, and WEP concentrations were all significantly affected by depth and land use (Table S1). WEN and WEP were especially low in all land uses, particularly below 30 cm (Table S6). Within the pasture and cropland, WEOC concentrations did not change with depth (*p* > 0.05). However, in the forest, WEOC decreased consistently with depth (*p* < 0.001). In the topsoil all three land uses differed from each other in WEOC concentrations, with forest containing the highest WEOC followed by pasture. WEN content similarly decreased with depth within each land use (Table S6), but not consistently through the profile, occurring at 30–40 cm in forest and cropland, and only at 60–70 cm in pasture (*p* < 0.001). The topsoil forest contained the highest WEN concentrations (35 mg kg^−1^) but below 10 cm this land use effect largely disappeared. Unlike WEOC and WEN, the effects of land use and depth on WEP only occurred in the lowest three soil depths, > 30–40 cm (Table S6; *p* < 0.05). In the pasture WEP concentrations were consistent throughout the soil profile. However, in the forest and cropland WEP decreased by more than half at the 60–70 cm depth (*p* < 0.05). Forest had the highest WEP content at 30–40 cm; however, > 60 cm, pasture had the highest WEP concentrations (*p* < 0.05).

### 3.8 Microbial Carbon Use Efficiency and Biomass C and N

Microbial CUE, measured only in the top and deepest soil layers, was affected by both land use and depth (Table S1). We observed a strong land use effect on CUE in the topsoil between the forest and cropland (*p* = 0.002), where cropland had the highest CUE (61.9%) and forest had the lowest (43.4%; Table S7). At the 85–100 cm depth there were no differences among land uses (Table S7; *p* > 0.05). While the deeper soils had higher CUE than the topsoil (*p* < 0.001), we suspect a methodological artifact given the high CUE values > 90%.

MBC and N was highly variable in our soils, likely masking seasonal, land use, and depth driven differences in our statistical analysis (Table S8). Forest was the only land use to show depth driven changes in MBC, with a decrease from topsoil (2060 mg kg^−1^; *p* < 0.001) to 30–40 cm (205 mg kg^−1^). MBC was also higher in the forest compared to the other land uses in the topsoil. MBN decreased at 30–40 cm in the forest (*p* < 0.001) and cropland (*p* < 0.001) but at the 60–70 cm depth in the pasture (*p* < 0.001). Forest also had higher MBN concentrations in the topsoil compared to cropland (*p* = 0.001), but pasture did not differ from either land use (*p* > 0.05).

## 4 Discussion

Our results reveal patterns in subsoil MAOM that largely contradict both our hypotheses, as well as prevailing theories about OM accumulation in subsoils. We expected variation in MAOM composition by depth and land use, primarily reflecting differences in OM inputs and its subsequent microbial processing. Yet, while topsoil followed typical land‐use driven patterns, below 30 cm, land‐use effects largely disappeared, especially between forest and cropland. We further anticipated seasonal shifts associated with time since flood exposure in WEOM and CUE, or even in MAOM due to rapid de/resorption dynamics under changing moisture conditions (Lieberman et al. 2023). However, seasonal effects on OM, moisture, and CUE were limited. Seasonal differences in OM inputs associated with litter inputs and harvest may have obscured early season flood‐related effects on WEOM and MAOM. Further, regardless of time since flooding, the soils were considerably wet. Water transport dynamics, mid‐season flooding associated with rainstorms, the generally high‐water table, and clay‐rich textures further complicate interpretations of soil moisture seasonal and depth dynamics. Thus, seasonal effects on MAOM inputs and persistence may not be immediately detectable at the field‐scale such that the depth‐driven trends we observed more likely reflect a history of flood exposure and not short‐term seasonal effects.

Lastly, we found that our soils exhibit depth trends contrary to current conceptual models and numerous experimental studies that emphasize how MAOM composition by depth is reflective of dominant inputs and formation processes (Guggenberger and Zech 1994; Hicks Pries et al. 2023; Kaiser et al. 2004; Kaiser and Kalbitz 2012; Lange et al. 2023; Leinemann et al. 2018; Liebmann et al. 2020; Pei et al. 2025; Rumpel and Kögel‐Knabner 2010; Sanderman et al. 2008). Rather, our findings suggest that in these poorly drained, flood‐exposed soils, subsoil MAOM composition is governed more by a compound's resistance to abiotic flood‐induced destabilization than by MAOM formation processes.

### 4.1 Land Use Differences in OM Are Primarily Constrained to the Topsoil

As expected, land use shaped topsoil C, N, and P concentrations, microbial biomass, and CUE. Higher aboveground OM inputs and reduced disturbance in the forest likely drove the higher soluble C and N and MAOM C, N, and P we observed (Chatterjee et al. 2018). We also found higher microbial biomass but a lower CUE in the forest compared to pasture and cropland. Yet, subsoil MAOM concentrations and composition are nearly identical between the forest and cropland. While pasture had distinct depth trends from forest and cropland, this does not seem to be caused by different topsoil processes, as differences became more pronounced in the subsoil. The muted land‐use effect on subsurface MAOM between forest and cropland is unexpected given that plant community composition is often a major factor in subsoil MAOM formation, especially the influence of roots (Heckman et al. 2023; Hobley et al. 2017; Lange et al. 2023; Xu et al. 2021). Yet, previous findings are based largely on well‐drained soils, and in contrast, Lei et al. (2025), similarly found that land use effects on subsoil total C, N, and P concentrations decrease within a floodplain.

Similarities in subsurface microbial activity may partially explain the convergence of MAOM composition and concentrations between the forest and cropland. MAOM depth trends are driven by repeated microbial transformations of downward‐moving OM (Kaiser and Kalbitz 2012; Leinemann et al. 2018; Liebmann et al. 2020). As surface OM is transported downward, particularly as WEOM, the expected lowering of OM C:N:P and enrichment of ^13^C is affected by the degree of resource limitations (i.e., WEOM) and microbial community CUE (Zechmeister‐Boltenstern et al. 2015). Yet, we found that despite topsoil differences, subsoil δ^13^C MAOM, CUE, MBC, and WEOM composition were similar between forest and cropland, indicating similar microbial activity and processing. Thus, the observed similarities in subsoil MAOM between the forest and cropland may reflect their convergence of microbial OM processing and subsequently the composition of OM entering subsoil MAOM at lower depths. It is worth noting that the exceptionally high δ^13^C signature of the forest and cropland subsoil cannot be fully explained by increased microbially processing or modern‐day C_4_ plant inputs in the cropland. We suspect buried aquatic plant inputs (δ^13^C −13% to −17‰) from the ancient fluvio‐lacustrine environment of the forest and cropland may be contributing to these enriched δ^13^C subsoil values (Hunt 1966). As the C age of the forest and cropland subsoil is quite old, as indicated by highly depleted Δ^14^C values, the soil is likely disproportionately composed of organic C derived from its past aquatic environments than from newer more ^13^C‐depleted terrestrial plant inputs. In contrast, the pasture is underlain by fluvio‐lacustrine silt to sandy clay loam and thus the δ^13^C may be less affected by aquatic plant inputs. Further, given the younger age of soil C in the pasture relative to the forest and cropland, δ^13^C values are also likely more strongly influenced by recent terrestrial plant‐derived inputs.

### 4.2 Resistance to Flood‐Induced Destabilization Likely Drives Subsoil MAOM Persistence

**FIGURE 5:**
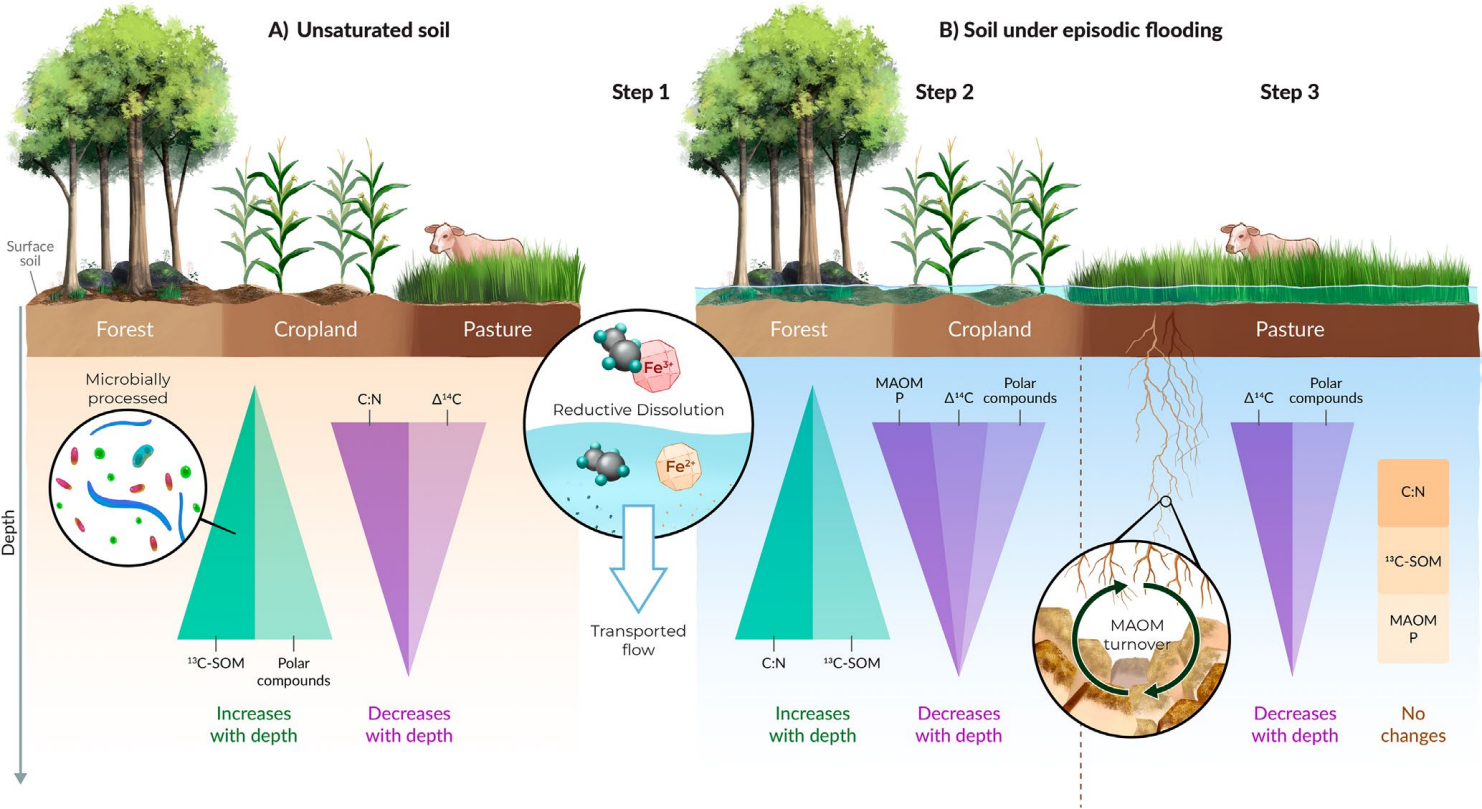
Proposed conceptual model of how OM moves, transforms and persists throughout the soil profile in our floodplain soils. In an unsaturated soil (A), certain theoretical depth dependent trends are expected across land uses, where microbially processed and polar compounds increase with depth, while C:N, C:P, and C age decrease with depth. Episodic or seasonal flooding (B) shifts these depth‐dependent trends as it simultaneously increases the downward transport of OM to the subsoil and induces reductive dissolution of Fe oxides, releasing MAOM attached (Step 1; B). As polar and/or N/P enriched compounds are more likely to form bonds with Fe oxides, they are disproportionally more affected by this reductive dissolution. These compounds are also more water soluble leading them to leach out of the soil once desorbed (Step 2; B). While non‐polar C rich compounds are resistant to loss with flooding, they are sensitive to destabilization induced by belowground inputs through root and microbial priming due to the relatively weak bonds they form within MAOM. The pasture in contrast to the forest and cropland system has extensive fine root biomass such that new belowground inputs can displace older non‐polar C rich microbially processed compounds. (Step 3; B). Thus, subsoil MAOM in the forest and cropland is composed of older, microbially processed C rich compounds, while the pasture MAOM is composed younger, plant derived P rich compounds. Original artwork by Elena Hartley (ELabArts.com).

We found that, contrary to current understandings of MAOM persistence and composition (Kleber et al. 2021; Lavallee et al. 2020), our subsoil forest and cropland MAOM and to an extent pasture MAOM consisted of relatively C‐rich (high C:N), insoluble, non‐polar compounds (high log K_ow_). Typically, MAOM persistence is attributed to formation pathways that favor polar and N/P enriched compounds that form strong mineral associations and thus preferentially accumulate and persist within MAOM (Jilling et al. 2018; Kleber et al. 2015; Lieberman et al. 2025). However, like any biogeochemical pool, MAOM concentration and composition is a net result of inputs and losses, and the compound properties that support MAOM formation are not necessarily the same as those leading to its loss. Here we suggest that in these flood‐prone systems, MAOM concentration and composition is better explained by compound properties affecting MAOM loss, that is, their sensitivity to flood‐induce destabilization. Based on our results, we present a conceptual framework describing how OM may move, transform, and persist through the soil profile of these soils undergoing periodic water saturation. With a two‐step destabilization process for our forest and cropland subsoils derived from the mineral surface and compound properties (Figure 5; Sections 4.2.1 and 4.2.2), and a three‐step destabilization process for the pasture (Figure 5; Section 4.2.3).

#### 4.2.1 Flood Induced Redox Changes Shift How Metals Impact MAOM Persistence

Fe–(oxyhydr) oxides are one of the strongest MAOM stabilizing agents under aerobic conditions (Gao et al. 2018; Mikutta et al. 2007; Saidy et al. 2013) and are often a significant predictor of MAOM stocks (Bramble et al. 2024; Rasmussen et al. 2018; von Fromm et al. 2025). Yet, in floodplains, Fe is susceptible to reductive dissolution under anoxic and reducing conditions, thus MAOM associated with Fe can be destabilized, rendering it a much weaker persistence mechanism (Figure 5, *Step 1*) (Maranguit et al. 2017; Yang et al. 2025). We found that while MAOM Fe concentrations varied by land use, they did not correspond to differences in MAOM concentrations among land uses.

In these reducing environments, other redox‐stable metals such as Ca may become more important predictors of MAOM persistence, especially in soils with higher pH conditions like ours (Rowley et al. 2018). Notably, in the pasture which had the lowest subsoil MAOM C, Ca concentrations were also low across all depths compared to the forest and cropland. This may indicate that Ca‐OM complexation becomes a more important persistence mechanism with flooding. Paradoxically, Ca is often considered an important predictor of soil OM persistence in arid systems, while Fe/Al exerts stronger control in wetter ecosystems (Heckman et al. 2023; Rasmussen et al. 2018; von Fromm et al. 2025). Yet, these and many other studies on MAOM processes systematically exclude floodplain or poorly‐drained soils, limiting the scope of their findings. In contrast, LaCroix et al. (2019) similarly found that Fe was a poor predictor of C content in the subsoil of a seasonal floodplain. While short range Fe minerals and exchangeable Ca that more specifically interact with OM were not captured in our study (Heckman et al. 2023; Liu et al. 2025), Fe remains redox sensitive and Ca redox insensitive. Thus, we suggest Fe's capacity to promote OM persistence follows a bell‐shaped curve with moisture, where Ca replaces Fe as a dominant MAOM control under both low and excessively high moisture, especially in higher soil pH conditions.

#### 4.2.2 Compound Characteristics Further Subvert Expectations of Depth‐Trends and Persistence

As expected, MAOM C and N concentrations (per g of soil) declined with depth within each land use, as did MAOM P in the forest. These decreases coincided with a relative decrease in soluble, polar compounds beginning at 30–40 cm in the forest and cropland and at 60–70 cm in the pasture. Typically, we would expect the reverse: As polar compounds, often enriched in N and P, form strong mineral and organo associations, especially with Fe (oxyhydr) oxides, they tend to preferentially accumulate in older, deeper MAOM (Rumpel et al. 2012). We posit that the affinity of polar compounds to Fe renders them disproportionately more affected by the reductive dissolution of Fe with flooding. Polar compounds are also typically more water soluble (Chiou and Kile 1994). Consequently, once desorbed from MAOM they can enter the aqueous phase and leach from the soil system. Hence, in our soils, the compound characteristics that typically enhance MAOM persistence in well‐drained soils may promote MAOM loss during a flood event, leading to a MAOM pool primarily composed of C rich, non‐polar compounds such as what we observed in our soils (Figure 5, *Step 2*).

Moreover, although considered less persistent in other soil systems, insoluble, non‐polar, C‐rich compounds accumulating at depth appear to be highly persistent within the context of our floodplain soil, since the subsoil MAOM is quite old (based on radiocarbon age) relative to well‐drained soils (Angst et al. 2018; Fontaine et al. 2007; Shi et al. 2020). While the ^14^C reflects MAOM's mean age, there clearly is a substantial portion of subsoil MAOM within each land use that demonstrates a high level of resistance to flood destabilization.

#### 4.2.3 Pasture MAOM Content and Composition Reflects Root Inputs and Fast Cycling MAOM

In the pasture we found a similar decrease in soluble, polar compounds and no correlation between Fe and MAOM content, suggesting susceptibility to the same selective desorption and loss as the forest and cropland. Yet, pasture MAOM diverged from forest and cropland in several significant ways: (1) C:N and δ^13^C remained stable with depth but shifted in the other land uses; (2) MAOM P remained at an elevated concentration, with a greater decrease in C:P compared to the other land uses in the lowest depths; (3) lignin relative abundance was very high at 85–100 cm; and (4) Δ^14^C was higher with depth compared to forest and cropland. These differences indicate the pasture subsoil MAOM contains younger and less decomposed plant contributions.

Unlike forest and croplands, grasslands have extensive fine root systems which are exceptionally important for MAOM accrual but may also displace a portion of the older subsoil MAOM (Sokol and Bradford 2019). While the insoluble, non‐polar C rich compounds accumulating at depth across all the land uses may be more resilient to flood destabilization, they still form relatively weak MAOM associations, typically via Van der Waal forces or hydrogen bonds (Kaiser and Kalbitz 2012; Kleber et al. 2021). These weak bonds leave the MAOM vulnerable to destabilization by other forces, like new root inputs that can desorb older compounds (Bird et al. 2008; Spohn and Schleuss 2019). We suspect the root inputs combined with legacy losses of some older subsoil MAOM from land use conversion in the pasture are contributing to the younger MAOM (Figure 5, *Step 3*). While croplands typically show lower subsoils MAOM C content due to fewer belowground inputs, our pasture contained the lowest MAOM C content > 60 cm and youngest MAOM (Georgiou et al. 2022; Hobley et al. 2017). Roots deeper in the soil profile can prime the decomposition of existing MAOM (Fontaine et al. 2007; Shahzad et al. 2018) and root exudate organic acids are well‐known MAOM destabilizers (Jilling et al. 2021; Keiluweit et al. 2015). Given pasture's lower subsurface MAOM C concentrations, the new root inputs are likely cycled out of MAOM relatively rapidly, preventing significant MAOM C accumulation (Jilling et al. 2025). Further, soil P can be a strong desorbing agent of MAOM C and N and outcompete C/N rich compounds for sorption sites, increasing MAOM P (Guppy et al. 2005; Spohn et al. 2022). Compared to the other land uses, pasture had higher MAOM P and soluble P and lower MAOM C:P (at > 60 cm), indicating potential P‐induced desorption. As such, the diverging depth trends we observed in pasture MAOM may reflect the dual role of roots and higher soluble P replacing older C‐rich compounds. Yet, these new root inputs are not necessarily persistent or high enough in concentration to yield the relatively higher MAOM C concentrations we would expect compared to croplands. Further, organo‐mineral P bonds are sensitive to flooding, and so the pasture MAOM may include some relatively fast cycling OM regularly replenished by belowground inputs (Lieberman et al. 2023, 2025). Our findings highlight the importance of MAOM composition in determining whether belowground inputs increase or decrease MAOM content, where weakly bound compounds may be more susceptible to loss with increased belowground inputs.

## 5 Conclusions and Implications

Our findings reveal that in seasonally flooded temperate soils, subsoil MAOM patterns diverge sharply from those more typically observed in well‐drained, drier soils. Notably, compound properties that enhance MAOM persistence in well‐drained soils, such as polarity and nutrients, appear to make MAOM more vulnerable to desorption and loss under saturated conditions. Instead, insoluble, non‐polar compounds that form weaker mineral associations seem to accumulate and persist under flood‐disturbed soils. We also found that land use effects, though evident in the topsoil, diminish with depth, likely because flooding overrides surface‐driven processes. However, pasture was an exception, showing distinct depth trends from forest and cropland. Here, even those compounds relatively resistant to flooding may be displaced by deep roots, leading to faster‐cycling and younger subsoil MAOM. Overall, our findings highlight that in flood‐prone environments, MAOM persistence is shaped more by compound resistance to hydrological stress than by formation pathways. As flooding increases globally (Hirabayashi et al. 2021), more subsoils could experience MAOM destabilization, releasing previously protected C. As such, we emphasize the urgent need to incorporate floodplain and poorly‐drained soils into conceptual frameworks, field studies, and empirical C models to improve predictions of subsoil MAOM dynamics under future climate change.

## Author Contributions

**Christian von Sperber:** conceptualization, supervision, resources, writing – review and editing. **Rachael Harman‐Denhoed:** methodology, writing – review and editing. **A. Stuart Grandy:** resources, writing – review and editing. **Hannah P Lieberman:** conceptualization, methodology, writing – original draft, writing – review and editing, visualization, investigation, funding acquisition. **Cynthia M Kallenbach:** writing – review and editing, conceptualization, resources, supervision, funding acquisition.

## Funding

This work was supported by Ministère de l'Agriculture, des Pêcheries et de l'Alimentation. Natural Sciences and Engineering Research Council of Canada (#528285, RGPIN‐2021‐03250). Fonds de recherche du Québec—Nature et technologies (316772).

## Conflicts of Interest

The authors declare no conflicts of interest.

## Supporting information


**Figure S1:** Location of sampling sites and the land use gradient. Soil samples were collected near Baie‐Du‐Febvre, Quebec, Canada (A) and included three different land uses (B): a cropland with a corn and soybean rotation under standard practices of the region (1); a native Maple‐dominated forest (2); and a managed pasture (3). Land‐use sites experience near‐annual spring flooding from Lake Saint Pierre and river tributaries (C), and May 2021 samples were collected two weeks after flood retreat (D). Images of land uses are forest, pasture, and cropland, left to right.
**Methods S1**. Stable isotope calculations.
**Methods S2**. Carbon use efficiency methodological details.
**Table S1:** Three‐way ANOVA results for land use by depth by season for soil moisture content (SMC; %), mineral‐associated (MAOM; g kg^−1^ dry soil) pools, MAOM δ^13^C and δ^15^N (‰), water‐extracted (WE; mg kg^−1^ dry soil) pools, microbial biomass C and N (MBC, MBN; mg kg^−1^ dry soil), and carbon use efficiency (CUE; %). Mineral‐associated δ^13^C was evaluated with a generalized linear model with a Gaussian distribution due to non‐normality. NS denotes no significance, * indicates significant at *p* < 0.05; ** indicates significance at *p* < 0.01, and *** indicate significance at *p* < 0.001. n = 4, Land use d.f. = 2; Depth d.f. = 3; Season d.f. = 1; Land use*Depth d.f. = 6; Land use*Season d.f. = 2; Depth*Season d.f. = 3; Land use*Depth*Season d.f. = 6.
**Table S2:** Mean ± standard error of soil moisture content (%) for forest, pasture, and cropland sites at 0–10, 30–40, 60–70, and 85–100 cm depths in spring and fall. Different uppercase letters indicate significant pairwise differences within one depth and season among land uses (*p* < 0.05); *n* = 4, *d.f*. = 2. Different lowercase letters indicate significant pairwise differences within one land use and season among depths (*p* < 0.05); *n* = 4, *d.f. =* 3. Asterisks indicate significant difference between seasons for the same land use and depth (*p* < 0.05); *n* = 4, *d.f. =* 3.
**Table S3:** Mean ± standard error of mineral‐associated N:P, and natural abundance isotope ratio of mineral‐associated δ^15^N (‰) for forest, pasture, and cropland sites at 0–10‐, 30–40‐, 60–70‐, and 85–100‐cm depths with seasons combined. Different uppercase letters indicate significant pairwise differences within one depth between land uses (*p* < 0.05); *n* = 8, d.f. = 2. Different lowercase letters indicate significant pairwise differences within one land use between depths (*p* < 0.05); *n = 8, d.f. = 3*.
**Table S4:** Two‐way ANOVA results for land use by depth for the relative abundance of each compound class (%) of mineral‐associated compounds and plant litter for forest, pasture, and cropland sites at 0–10‐, 30–40‐, 60–70‐ and 85–100‐cm depths in the spring. NS denotes no significance, * indicates significant at *p* < 0.05, ** indicates significance at *p* < 0.01, and *** indicate significance at *p* < 0.001. n = 4, Land use d.f. = 2; Depth d.f. = 4; Land use*Depth d.f. = 8.
**Table S5:** Two‐way ANOVA results for land use by depth for the relative abundance (%) of mineral‐associated compounds by log K_ow_ level for forest, pasture, cropland at 0–10‐, 30–40‐, 60–70 and 85–100‐cm depths during the spring. NS denotes no significance, * indicates significant at *p* < 0.05, ** indicates significance at *p* < 0.01, and *** indicate significance at *p* < 0.001. n = 4, Land use d.f. = 2; Depth d.f. = 3; Land use*Depth d.f. = 6.
**Table S6:** Mean ± standard error of water‐extracted organic C (WEOC; mg kg^−1^ dry soil), water‐extracted N (WEN; mg kg^−1^ dry soil), water‐extracted inorganic P (WEP; mg kg^−1^ dry soil), water‐extracted C:N (WE‐C:N), water‐extracted C:P (WE‐C:P), and water‐extracted N:P (WE‐N:P) for forest, pasture, and cropland sites at 0–10‐, 30–40‐, 60–70‐, and 85–100‐cm depths with seasons combined. Different uppercase letters indicate significant pairwise differences within one depth among land uses (*p* < 0.05); *n* = 8, *d.f. =* 2. Different lowercase letters indicate significant pairwise differences within one land use among depths (*p* < 0.05); *n* = 8, *d.f. =* 3.
**Table S7:** Mean ± standard error of carbon use efficiency (%) for forest, pasture, and cropland sites at 0–10 and 85–100‐cm depths with seasons combined. Different uppercase letters indicate significant pairwise differences within one depth among land uses (*p* < 0.05); *n* = 8, *d.f. =* 2. Different lower‐case letters indicate significant pairwise differences within one land use among depths (*p* < 0.05); *n* = 8, *d.f. =* 3.
**Table S8:** Mean ± standard error microbial biomass C (MBC; mg kg^−1^ dry soil) and microbial biomass N (MBN; mg kg^−1^ dry soil) for forest, pasture, and cropland sites at 0–10‐, 30–40‐, 60–70‐, and 85–100‐cm depths with seasons combined. Due to limited sample, we were unable to conduct these analyses on MBC in the Forest at 0–10 cm for the fall. Different uppercase letters indicate significant pairwise differences within one depth among land uses (*p* < 0.05); *n* = 8, *d.f. =* 2. Different lowercase letters indicate significant pairwise differences within one land use among depths (*p* < 0.05); *n* = 8, *d.f. =* 3.

## Data Availability

All data from this study were deposited and freely available at https://doi.org/10.5683/SP3/9M8JYJ Borealis McGill University Dataverse Collection.

## References

[gcb70860-bib-0001] Angers, D. A. , M. H. Chantigny , J. D. MacDonald , P. Rochette , and D. Côté . 2010. “Differential Retention of Carbon, Nitrogen and Phosphorus in Grassland Soil Profiles With Long‐Term Manure Application.” Nutrient Cycling in Agroecosystems 86, no. 2: 225–229.

[gcb70860-bib-0002] Angst, G. , J. Messinger , M. Greiner , et al. 2018. “Soil Organic Carbon Stocks in Topsoil and Subsoil Controlled by Parent Material, Carbon Input in the Rhizosphere, and Microbial‐Derived Compounds.” Soil Biology and Biochemistry 122: 19–30.

[gcb70860-bib-0003] Annex, C. 2008. Stockholm Convention on Persistent Organic Pollutants. Vol. 29. United Nations Environmental Programme.

[gcb70860-bib-0004] Bai, Y. , and M. F. Cotrufo . 2022. “Grassland Soil Carbon Sequestration: Current Understanding, Challenges, and Solutions.” Science 377, no. 6606: 603–608. 35926033 10.1126/science.abo2380

[gcb70860-bib-0005] Bailey, V. L. , C. Hicks Pries , and K. Lajtha . 2019. “What Do We Know About Soil Carbon Destabilization?” Environmental Research Letters 14, no. 8: 083004.

[gcb70860-bib-0006] Baldock, J. A. , J. Sanderman , L. M. Macdonald , et al. 2013. “Quantifying the Allocation of Soil Organic Carbon to Biologically Significant Fractions.” Soil Research 51, no. 8: 561–576.

[gcb70860-bib-0007] Balesdent, J. , I. Basile‐Doelsch , J. Chadoeuf , et al. 2018. “Atmosphere–Soil Carbon Transfer as a Function of Soil Depth.” Nature 559, no. 7715: 599–602. 29995858 10.1038/s41586-018-0328-3

[gcb70860-bib-0008] Bird, J. A. , M. Kleber , and M. S. Torn . 2008. “13C and 15N Stabilization Dynamics in Soil Organic Matter Fractions During Needle and Fine Root Decomposition.” Organic Geochemistry 39, no. 4: 465–477. 10.1016/j.orggeochem.2007.12.003.

[gcb70860-bib-0009] Bramble, D. S. E. , S. Ulrich , I. Schöning , et al. 2024. “Formation of Mineral‐Associated Organic Matter in Temperate Soils Is Primarily Controlled by Mineral Type and Modified by Land Use and Management Intensity.” Global Change Biology 30, no. 1: e17024. 37986273 10.1111/gcb.17024

[gcb70860-bib-0010] Bridge, J. S. 2009. Rivers and Floodplains: Forms, Processes, and Sedimentary Record. John Wiley & Sons.

[gcb70860-bib-0011] Campeau, S. , J. Ruiz , B. Bourgeois , et al. 2024. Pôle d'expertise multidisciplinaire en gestion durable du littoral du lac Saint‐Pierre, rapport final 2019–2024.

[gcb70860-bib-0012] Chatterjee, N. , P. K. Ramachandran Nair , S. Chakraborty , and V. D. Nair . 2018. “Changes in Soil Carbon Stocks Across the Forest‐Agroforest‐Agriculture/Pasture Continuum in Various Agroecological Regions: A Meta‐Analysis.” Agriculture, Ecosystems & Environment 266: 55–67.

[gcb70860-bib-0013] Chen, H. , C. C. Rhoades , and A. T. Chow . 2020. “Characteristics of Soil Organic Matter 14 Years After a Wildfire: A Pyrolysis‐Gas‐Chromatography Mass Spectrometry (Py‐GC‐MS) Study.” Journal of Analytical and Applied Pyrolysis 152: 104922.

[gcb70860-bib-0014] Chiou, C. T. , and D. E. Kile . 1994. “Effects of Polar and Nonpolar Groups on the Solubility of Organic Compounds in Soil Organic Matter.” Environmental Science & Technology 28, no. 6: 1139–1144. 22176242 10.1021/es00055a026

[gcb70860-bib-0015] Dauphin, D. , and B. Jobin . 2016. “Changements de l'occupation du sol dans la plaine inondable du lac Saint‐Pierre entre les années 1950 et 1997.” Le Naturaliste Canadien 140, no. 1: 42–52.

[gcb70860-bib-0016] Dictor, M.‐C. , L. Tessier , and G. Soulas . 1998. “Reassessement of the KEC Coefficient of the Fumigation–Extraction Method in a Soil Profile.” Soil Biology and Biochemistry 30, no. 2: 119–127.

[gcb70860-bib-0019] Fierer, N. , and J. P. Schimel . 2003. “A Proposed Mechanism for the Pulse in Carbon Dioxide Production Commonly Observed Following the Rapid Rewetting of a Dry Soil.” Soil Science Society of America Journal 67, no. 3: 798–805.

[gcb70860-bib-0020] Fontaine, S. , S. Barot , P. Barré , N. Bdioui , B. Mary , and C. Rumpel . 2007. “Stability of Organic Carbon in Deep Soil Layers Controlled by Fresh Carbon Supply.” Nature 450, no. 7167: 277–280. 17994095 10.1038/nature06275

[gcb70860-bib-0021] Fröberg, M. , P. M. Jardine , P. J. Hanson , et al. 2007. “Low Dissolved Organic Carbon Input From Fresh Litter to Deep Mineral Soils.” Soil Science Society of America Journal 71, no. 2: 347–354.

[gcb70860-bib-0022] Gao, J. , B. Jansen , C. Cerli , et al. 2018. “Organic Matter Coatings of Soil Minerals Affect Adsorptive Interactions With Phenolic and Amino Acids.” European Journal of Soil Science 69, no. 4: 613–624.

[gcb70860-bib-0023] Georgiou, K. , R. B. Jackson , O. Vindušková , et al. 2022. “Global Stocks and Capacity of Mineral‐Associated Soil Organic Carbon.” Nature Communications 13, no. 1: 3797. 10.1038/s41467-022-31540-9 PMC9249731 35778395

[gcb70860-bib-0024] Gimeno, S. , D. Allan , K. Paul , P. Remuzat , and M. Collard . 2024. “Are Current Regulatory Log Kow Cut‐Off Values Fit‐For‐Purpose as a Screening Tool for Bioaccumulation Potential in Aquatic Organisms?” Regulatory Toxicology and Pharmacology 147: 105556. 38158033 10.1016/j.yrtph.2023.105556

[gcb70860-bib-0025] Guggenberger, G. , and W. Zech . 1994. “Composition and Dynamics of Dissolved Carbohydrates and Lignin‐Degradation Products in Two Coniferous Forests, NE Bavaria, Germany.” Soil Biology and Biochemistry 26, no. 1: 19–27.

[gcb70860-bib-0026] Guppy, C. N. , N. W. Menzies , P. W. Moody , and F. P. C. Blamey . 2005. “Competitive Sorption Reactions Between Phosphorus and Organic Matter in Soil: A Review.” Soil Research 43, no. 2: 189–202. 10.1071/sr04049.

[gcb70860-bib-0027] Haines, W. B. 1930. “Studies in the Physical Properties of Soil. V. The Hysteresis Effect in Capillary Properties, and the Modes of Moisture Distribution Associated Therewith.” Journal of Agricultural Science 20, no. 1: 97–116.

[gcb70860-bib-0028] Harris, D. , W. R. Horwáth , and C. Van Kessel . 2001. “Acid Fumigation of Soils to Remove Carbonates Prior to Total Organic Carbon or Carbon‐13 Isotopic Analysis.” Soil Science Society of America Journal 65, no. 6: 1853–1856.

[gcb70860-bib-0029] Harrison, R. B. , P. W. Footen , and B. D. Strahm . 2011. “Deep Soil Horizons: Contribution and Importance to Soil Carbon Pools and in Assessing Whole‐Ecosystem Response to Management and Global Change.” Forest Science 57, no. 1: 67–76.

[gcb70860-bib-0030] Heckman, K. A. , A. R. Possinger , B. D. Badgley , et al. 2023. “Moisture‐Driven Divergence in Mineral‐Associated Soil Carbon Persistence.” Proceedings of the National Academy of Sciences 120, no. 7: e2210044120. 10.1073/pnas.2210044120 PMC9962923 36745807

[gcb70860-bib-0031] Hicks Pries, C. E. , C. Castanha , R. C. Porras , and M. S. Torn . 2017. “The Whole‐Soil Carbon Flux in Response to Warming.” Science 355, no. 6332: 1420–1423. 28280251 10.1126/science.aal1319

[gcb70860-bib-0032] Hicks Pries, C. E. , R. Ryals , B. Zhu , et al. 2023. “The Deep Soil Organic Carbon Response to Global Change.” Annual Review of Ecology, Evolution, and Systematics 54, no. 1: 375–401.

[gcb70860-bib-0033] Hirabayashi, Y. , M. Tanoue , O. Sasaki , X. Zhou , and D. Yamazaki . 2021. “Global Exposure to Flooding From the New CMIP6 Climate Model Projections.” Scientific Reports 11, no. 1: 3740. 33580166 10.1038/s41598-021-83279-w PMC7881105

[gcb70860-bib-0034] Hobley, E. , J. Baldock , Q. Hua , and B. Wilson . 2017. “Land‐Use Contrasts Reveal Instability of Subsoil Organic Carbon.” Global Change Biology 23, no. 2: 955–965. 27252113 10.1111/gcb.13379

[gcb70860-bib-0035] Hudon, C. , M. Jean , and G. Létourneau . 2018. “Temporal (1970–2016) Changes in Human Pressures and Wetland Response in the St. Lawrence River (Québec, Canada).” Science of the Total Environment 643: 1137–1151. 30189531 10.1016/j.scitotenv.2018.06.080

[gcb70860-bib-0036] Hunt, J. M. 1966. The Significance of Carbon Isotope Variations in Marine Sediments. Advances in Organic Geochemistry. Proceedings of the Third International Congress, London.

[gcb70860-bib-0037] Jean, M. , and G. Létourneau . 2011. “Changes to the Wetlands of the St. Lawrence River From 1970 to 2002.” In, edited by Environment Canada, Science and Technology Branch, Water Quality Monitoring .

[gcb70860-bib-0038] Jilling, A. , A. S. Grandy , A. B. Daly , et al. 2025. “Evidence for the Existence and Ecological Relevance of Fast‐Cycling Mineral‐Associated Organic Matter.” Communications Earth & Environment 6, no. 1: 690.

[gcb70860-bib-0039] Jilling, A. , D. Kane , A. Williams , et al. 2020. “Rapid and Distinct Responses of Particulate and Mineral‐Associated Organic Nitrogen to Conservation Tillage and Cover Crops.” Geoderma 359. 10.1016/j.geoderma.2019.114001.

[gcb70860-bib-0040] Jilling, A. , M. Keiluweit , A. R. Contosta , et al. 2018. “Minerals in the Rhizosphere: Overlooked Mediators of Soil Nitrogen Availability to Plants and Microbes.” Biogeochemistry 139, no. 2: 103–122. 10.1007/s10533-018-0459-5.

[gcb70860-bib-0041] Jilling, A. , M. Keiluweit , J. L. M. Gutknecht , and A. S. Grandy . 2021. “Priming Mechanisms Providing Plants and Microbes Access to Mineral‐Associated Organic Matter.” Soil Biology and Biochemistry 158: 108265.

[gcb70860-bib-0042] Jobbágy, E. G. , and R. B. Jackson . 2000. “The Vertical Distribution of Soil Organic Carbon and Its Relation to Climate and Vegetation.” Ecological Applications 10, no. 2: 423–436.

[gcb70860-bib-0043] Jobin, B. , and P. Brodeur . 2023. “Changements de l'occupation du sol de la plaine inondable du lac Saint‐Pierre de 1950 à 2016 et perspectives pour la restauration des milieux naturels.” Le Naturaliste Canadien 147, no. 2: 14–26.

[gcb70860-bib-0044] Kaiser, K. , G. Guggenberger , and L. Haumaier . 2004. “Changes in Dissolved Lignin‐Derived Phenols, Neutral Sugars, Uronic Acids, and Amino Sugars With Depth in Forested Haplic Arenosols and Rendzic Leptosols.” Biogeochemistry 70: 135–151.

[gcb70860-bib-0045] Kaiser, K. , and K. Kalbitz . 2012. “Cycling Downwards – Dissolved Organic Matter in Soils.” Soil Biology and Biochemistry 52: 29–32. 10.1016/j.soilbio.2012.04.002.

[gcb70860-bib-0046] Kallenbach, C. M. , A. S. Grandy , S. D. Frey , and A. F. Diefendorf . 2015. “Microbial Physiology and Necromass Regulate Agricultural Soil Carbon Accumulation.” Soil Biology and Biochemistry 91: 279–290.

[gcb70860-bib-0047] Keiluweit, M. , J. J. Bougoure , P. S. Nico , J. Pett‐Ridge , P. K. Weber , and M. Kleber . 2015. “Mineral Protection of Soil Carbon Counteracted by Root Exudates.” Nature Climate Change 5, no. 6: 588–595. 10.1038/nclimate2580.

[gcb70860-bib-0048] Kim, S. , J. Chen , T. Cheng , et al. 2025. “PubChem 2025 Update.” Nucleic Acids Research 53, no. D1: D1516–D1525. 39558165 10.1093/nar/gkae1059 PMC11701573

[gcb70860-bib-0049] Kleber, M. , I. C. Bourg , E. K. Coward , C. M. Hansel , S. C. Myneni , and N. Nunan . 2021. “Dynamic Interactions at the Mineral–Organic Matter Interface.” Nature Reviews Earth & Environment 2, no. 6: 402–421.

[gcb70860-bib-0050] Kleber, M. , K. Eusterhues , M. Keiluweit , C. Mikutta , R. Mikutta , and P. S. Nico . 2015. “Mineral–Organic Associations: Formation, Properties, and Relevance in Soil Environments.” Advances in Agronomy 130: 1–140.

[gcb70860-bib-0051] LaCroix, R. E. , M. M. Tfaily , M. McCreight , M. E. Jones , L. Spokas , and M. Keiluweit . 2019. “Shifting Mineral and Redox Controls on Carbon Cycling in Seasonally Flooded Mineral Soils.” Biogeosciences 16, no. 13: 2573–2589.

[gcb70860-bib-0052] Land‐Miller, H. , A. M. Roos , M. Simon , et al. 2024. “Comparison of Feeding Niches Between Arctic and Northward‐Moving Sub‐Arctic Marine Mammals in Greenland.” Marine Ecology Progress Series 728: 163–182.

[gcb70860-bib-0053] Lange, M. , N. Eisenhauer , H. Chen , and G. Gleixner . 2023. “Increased Soil Carbon Storage Through Plant Diversity Strengthens With Time and Extends Into the Subsoil.” Global Change Biology 29, no. 9: 2627–2639. 36799509 10.1111/gcb.16641

[gcb70860-bib-0054] Lavallee, J. M. , J. L. Soong , and M. F. Cotrufo . 2020. “Conceptualizing Soil Organic Matter Into Particulate and Mineral‐Associated Forms to Address Global Change in the 21st Century.” Global Change Biology 26, no. 1: 261–273. 31587451 10.1111/gcb.14859

[gcb70860-bib-0055] Lehmann, J. , C. M. Hansel , C. Kaiser , et al. 2020. “Persistence of Soil Organic Carbon Caused by Functional Complexity.” Nature Geoscience 13, no. 8: 529–534. 10.1038/s41561-020-0612-3.

[gcb70860-bib-0056] Lei, K. , F. B. Bucka , C. Just , et al. 2025. “Distinct Impact of Land Use and Soil Development Processes on Coupled Biogeochemical Cycling of C, N and P in a Temperate Hillslope‐Flood Plain System.” Biogeochemistry 168, no. 1: 21.

[gcb70860-bib-0057] Leinemann, T. , S. Preusser , R. Mikutta , et al. 2018. “Multiple Exchange Processes on Mineral Surfaces Control the Transport of Dissolved Organic Matter Through Soil Profiles.” Soil Biology and Biochemistry 118: 79–90.

[gcb70860-bib-0058] Lieberman, H. P. , M. Rothman , C. von Sperber , and C. M. Kallenbach . 2023. “Experimental Flooding Shifts Carbon, Nitrogen, and Phosphorus Pool Distribution and Microbial Activity.” Biogeochemistry 165, no. 1: 75–90.

[gcb70860-bib-0059] Lieberman, H. P. , C. von Sperber , and C. M. Kallenbach . 2025. “Soil Phosphorus Dynamics Are an Overlooked but Dominant Control on Mineral‐Associated Organic Matter.” Global Change Biology 31, no. 7: e70307. 40621656 10.1111/gcb.70307 PMC12230779

[gcb70860-bib-0060] Liebmann, P. , P. Wordell‐Dietrich , K. Kalbitz , et al. 2020. “Relevance of Aboveground Litter for Soil Organic Matter Formation—A Soil Profile Perspective.” Biogeosciences 17, no. 12: 3099–3113.

[gcb70860-bib-0061] Lin, Y. , A. Bhattacharyya , A. N. Campbell , P. S. Nico , J. Pett‐Ridge , and W. L. Silver . 2018. “Phosphorus Fractionation Responds to Dynamic Redox Conditions in a Humid Tropical Forest Soil.” Journal of Geophysical Research: Biogeosciences 123, no. 9: 3016–3027.

[gcb70860-bib-0062] Liu, T. , X. Wang , S. Wang , E. Zhu , S. J. Hall , and X. Feng . 2025. “Iron‐Driven Fast Decomposition of Soil Carbon Under Periodic Anoxia.” Global Change Biology 31, no. 4: e70184. 40231371 10.1111/gcb.70184

[gcb70860-bib-0063] Luo, Z. , E. Wang , and O. J. Sun . 2010. “Can No‐Tillage Stimulate Carbon Sequestration in Agricultural Soils? A Meta‐Analysis of Paired Experiments.” Agriculture, Ecosystems & Environment 139, no. 1–2: 224–231.

[gcb70860-bib-0064] Manzoni, S. , P. Taylor , A. Richter , A. Porporato , and G. I. Ågren . 2012. “Environmental and Stoichiometric Controls on Microbial Carbon‐Use Efficiency in Soils.” New Phytologist 196, no. 1: 79–91. 22924405 10.1111/j.1469-8137.2012.04225.x

[gcb70860-bib-0065] Maranguit, D. , T. Guillaume , and Y. Kuzyakov . 2017. “Effects of Flooding on Phosphorus and Iron Mobilization in Highly Weathered Soils Under Different Land‐Use Types: Short‐Term Effects and Mechanisms.” Catena 158: 161–170. 10.1016/j.catena.2017.06.023.

[gcb70860-bib-0066] McDowell, W. H. , and G. E. Likens . 1988. “Origin, Composition, and Flux of Dissolved Organic Carbon in the Hubbard Brook Valley.” Ecological Monographs 58, no. 3: 177–195.

[gcb70860-bib-0067] Mikutta, R. , C. Mikutta , K. Kalbitz , T. Scheel , K. Kaiser , and R. Jahn . 2007. “Biodegradation of Forest Floor Organic Matter Bound to Minerals via Different Binding Mechanisms.” Geochimica et Cosmochimica Acta 71, no. 10: 2569–2590.

[gcb70860-bib-0068] Mikutta, R. , S. Turner , A. Schippers , et al. 2019. “Microbial and Abiotic Controls on Mineral‐Associated Organic Matter in Soil Profiles Along an Ecosystem Gradient.” Scientific Reports 9, no. 1: 10294. 10.1038/s41598-019-46501-4. 31312015 PMC6635608

[gcb70860-bib-0069] Ofiti, N. O. E. , C. U. Zosso , J. L. Soong , et al. 2021. “Warming Promotes Loss of Subsoil Carbon Through Accelerated Degradation of Plant‐Derived Organic Matter.” Soil Biology and Biochemistry 156: 108185.

[gcb70860-bib-0070] Ohno, T. , and L. M. Zibilske . 1991. “Determination of Low Concentrations of Phosphorus in Soil Extracts Using Malachite Green.” Soil Science Society of America Journal 55, no. 3: 892–895.

[gcb70860-bib-0071] Patel, K. F. , A. Myers‐Pigg , B. Bond‐Lamberty , et al. 2021. “Soil Carbon Dynamics During Drying vs. Rewetting: Importance of Antecedent Moisture Conditions.” Soil Biology and Biochemistry 156: 108165.

[gcb70860-bib-0072] Pei, J. , J. Li , Y. Luo , et al. 2025. “Patterns and Drivers of Soil Microbial Carbon Use Efficiency Across Soil Depths in Forest Ecosystems.” Nature Communications 16, no. 1: 5218. 10.1038/s41467-025-60594-8 PMC12141608 40473650

[gcb70860-bib-0073] Rasmussen, C. , K. Heckman , W. R. Wieder , et al. 2018. “Beyond Clay: Towards an Improved Set of Variables for Predicting Soil Organic Matter Content.” Biogeochemistry 137: 297–306.

[gcb70860-bib-0074] Rowley, M. C. , S. Grand , and É. P. Verrecchia . 2018. “Calcium‐Mediated Stabilisation of Soil Organic Carbon.” Biogeochemistry 137, no. 1: 27–49.

[gcb70860-bib-0075] Rumpel, C. , A. Chabbi , and B. Marschner . 2012. “Carbon Storage and Sequestration in Subsoil Horizons: Knowledge, Gaps and Potentials.” In Recarbonization of the Biosphere: Ecosystems and the Global Carbon Cycle, 445–464. Springer.

[gcb70860-bib-0076] Rumpel, C. , and I. Kögel‐Knabner . 2010. “Deep Soil Organic Matter—A Key but Poorly Understood Component of Terrestrial C Cycle.” Plant and Soil 338, no. 1–2: 143–158. 10.1007/s11104-010-0391-5.

[gcb70860-bib-0077] Saidy, A. R. , R. J. Smernik , J. A. Baldock , K. Kaiser , and J. Sanderman . 2013. “The Sorption of Organic Carbon Onto Differing Clay Minerals in the Presence and Absence of Hydrous Iron Oxide.” Geoderma 209: 15–21.

[gcb70860-bib-0078] Samson, M.‐E. , M. H. Chantigny , A. Vanasse , S. Menasseri‐Aubry , I. Royer , and D. A. Angers . 2021. “Response of Subsurface C and N Stocks Dominates the Whole‐Soil Profile Response to Agricultural Management Practices in a Cool, Humid Climate.” Agriculture, Ecosystems & Environment 320: 107590.

[gcb70860-bib-0079] Sanderman, J. , J. A. Baldock , and R. Amundson . 2008. “Dissolved Organic Carbon Chemistry and Dynamics in Contrasting Forest and Grassland Soils.” Biogeochemistry 89, no. 2: 181–198.

[gcb70860-bib-0080] Schimel, J. P. , J. Å. Martin Wetterstedt , P. A. Holden , and S. E. Trumbore . 2011. “Drying/Rewetting Cycles Mobilize Old C From Deep Soils From a California Annual Grassland.” Soil Biology & Biochemistry 43, no. 5: 1101–1103. 10.1016/j.soilbio.2011.01.008.

[gcb70860-bib-0081] Shahzad, T. , M. I. Rashid , V. Maire , et al. 2018. “Root Penetration in Deep Soil Layers Stimulates Mineralization of Millennia‐Old Organic Carbon.” Soil Biology and Biochemistry 124: 150–160.

[gcb70860-bib-0082] Shi, Z. , S. D. Allison , Y. He , et al. 2020. “The Age Distribution of Global Soil Carbon Inferred From Radiocarbon Measurements.” Nature Geoscience 13, no. 8: 555–559.

[gcb70860-bib-0083] Sokol, N. W. , and M. A. Bradford . 2019. “Microbial Formation of Stable Soil Carbon Is More Efficient From Belowground Than Aboveground Input.” Nature Geoscience 12, no. 1: 46–53.

[gcb70860-bib-0084] Sokol, N. W. , E. D. Whalen , A. Jilling , C. M. Kallenbach , J. Pett‐Ridge , and K. Georgiou . 2022. “Global Distribution, Formation and Fate of Mineral‐Associated Soil Organic Matter Under a Changing Climate: A Trait‐Based Perspective.” Functional Ecology 36: 1411–1429.

[gcb70860-bib-0085] Soong, J. L. , C. Castanha , C. E. Hicks Pries , et al. 2021. “Five Years of Whole‐Soil Warming Led to Loss of Subsoil Carbon Stocks and Increased CO2 Efflux.” Science Advances 7, no. 21: eabd1343. 34020943 10.1126/sciadv.abd1343 PMC8139586

[gcb70860-bib-0086] Souza, L. F. T. , D. R. Hirmas , P. L. Sullivan , et al. 2023. “Root Distributions, Precipitation, and Soil Structure Converge to Govern Soil Organic Carbon Depth Distributions.” Geoderma 437: 116569.

[gcb70860-bib-0087] Spohn, M. , K. Diáková , F. Aburto , S. Doetterl , and J. Borovec . 2022. “Sorption and Desorption of Organic Matter in Soils as Affected by Phosphate.” Geoderma 405: 115377.

[gcb70860-bib-0088] Spohn, M. , and P.‐M. Schleuss . 2019. “Addition of Inorganic Phosphorus to Soil Leads to Desorption of Organic Compounds and Thus to Increased Soil Respiration.” Soil Biology and Biochemistry 130: 220–226.

[gcb70860-bib-0089] Sulman, B. N. , J. Harden , Y. He , et al. 2020. “Land Use and Land Cover Affect the Depth Distribution of Soil Carbon: Insights From a Large Database of Soil Profiles.” Frontiers in Environmental Science 8: 146.

[gcb70860-bib-0090] Tessier, L. , E. G. Gregorich , and E. Topp . 1998. “Spatial Variability of Soil Microbial Biomass Measured by the Fumigation Extraction Method, and KEC as Affected by Depth and Manure Application.” Soil Biology and Biochemistry 30, no. 10–11: 1369–1377.

[gcb70860-bib-0091] Tiessen, H. , and J. W. B. Stewart . 1983. “Particle‐Size Fractions and Their Use in Studies of Soil Organic Matter: II. Cultivation Effects on Organic Matter Composition in Size Fractions.” Soil Science Society of America Journal 47, no. 3: 509–514.

[gcb70860-bib-0092] Tipping, E. , P. M. Chamberlain , M. Fröberg , P. J. Hanson , and P. M. Jardine . 2012. “Simulation of Carbon Cycling, Including Dissolved Organic Carbon Transport, in Forest Soil Locally Enriched With 14C.” Biogeochemistry 108, no. 1: 91–107.

[gcb70860-bib-0093] Trumbore, S. E. , S. L. Schiff , R. Aravena , and R. Elgood . 1992. “Sources and Transformation of Dissolved Organic Carbon in the Harp Lake Forested Catchment: The Role of Soils.” Radiocarbon 34, no. 3: 626–635.

[gcb70860-bib-0018] U.S. EPA . 2007. “Method 3051A (SW‐846): Microwave Assisted Acid Digestion of Sediments, Sludges, and Oils,” Revision 1. Washington, DC.

[gcb70860-bib-0017] U.S. EPA . 2014. Method 6010D (SW‐846): Inductively Coupled Plasma‐Atomic Emission Spectrometry, Revision 4. Washington, DC.

[gcb70860-bib-0094] von Fromm, S. F. , A. M. Hoyt , C. A. Sierra , K. Georgiou , S. Doetterl , and S. E. Trumbore . 2024. “Controls and Relationships of Soil Organic Carbon Abundance and Persistence Vary Across Pedo‐Climatic Regions.” Global Change Biology 30, no. 5: e17320. 38751310 10.1111/gcb.17320

[gcb70860-bib-0095] von Fromm, S. F. , H. F. Jungkunst , B. Amenkhienan , et al. 2025. “Moisture and Soil Depth Govern Relationships Between Soil Organic Carbon and Oxalate‐Extractable Metals at the Global Scale.” Biogeochemistry 168, no. 1: 20.

[gcb70860-bib-0096] Wacker, L. , M. Christl , and H.‐A. Synal . 2010. “Bats: A New Tool for AMS Data Reduction.” Nuclear Instruments and Methods in Physics Research Section B: Beam Interactions with Materials and Atoms 268, no. 7–8: 976–979.

[gcb70860-bib-0097] Wu, J. , R. G. Joergensen , B. Pommerening , R. Chaussod , and P. C. Brookes . 1990. “Measurement of Soil Microbial Biomass C by Fumigation‐Extraction‐An Automated Procedure.” Soil Biology & Biochemistry 22, no. 8: 1167–1169.

[gcb70860-bib-0098] Xiang, S.‐R. , A. Doyle , P. A. Holden , and J. P. Schimel . 2008. “Drying and Rewetting Effects on C and N Mineralization and Microbial Activity in Surface and Subsurface California Grassland Soils.” Soil Biology and Biochemistry 40, no. 9: 2281–2289.

[gcb70860-bib-0099] Xu, S. , E. J. Sayer , N. Eisenhauer , X. Lu , J. Wang , and C. Liu . 2021. “Aboveground Litter Inputs Determine Carbon Storage Across Soil Profiles: A Meta‐Analysis.” Plant and Soil 462, no. 1: 429–444.

[gcb70860-bib-0100] Yang, L. , Y. Zou , Z. Jia , et al. 2025. “Iron Reduction Promotes Carbon Mineralization and Nutrient Release of Iron‐Associated Organic Matter in Anoxic Environments.” Water Research 284: 124032. 40541090 10.1016/j.watres.2025.124032

[gcb70860-bib-0101] Zechmeister‐Boltenstern, S. , K. M. Keiblinger , M. Mooshammer , et al. 2015. “The Application of Ecological Stoichiometry to Plant–Microbial–Soil Organic Matter Transformations.” Ecological Monographs 85, no. 2: 133–155.

