# Supplementary material for: Seasonal Flooding Disrupts Expected Depth‐Dependent Patterns of Mineral Associated Carbon, Nitrogen, and Phosphorus Across Land Uses
Source: Glob Chang Biol. 2026 Apr 17;32(4):e70860. doi: 10.1111/gcb.70860 (PMC13088760; doi:10.1111/gcb.70860)
Supplement: Supplementary file 1 — Figure S1: Location of sampling sites and the land use gradient. Soil samples were collected near Baie‐Du‐Febvre, Quebec, Canada (A) and included three different land uses (B): a cropland with a corn and soybean rotation under standard practices of the region (1); a native Maple‐dominated forest (2); and a managed pasture (3). Land‐use sites experience near‐annual spring flooding from Lake Saint Pierre and river tributaries (C), and May 2021 samples were collected two weeks after flood retreat (D). Images of land uses are forest, pasture, and cropland, left to right. Methods S1. Stable isotope calculations. Methods S2. Carbon use efficiency methodological details. Table S1: Three‐way ANOVA results for land use by depth by season for soil moisture content (SMC; %), mineral‐associated (MAOM; g kg−1 dry soil) pools, MAOM δ13C and δ15N (‰), water‐extracted (WE; mg kg−1 dry soil) pools, microbial biomass C and N (MBC, MBN; mg kg−1 dry soil), and carbon use efficiency (CUE; %). Mineral‐associated δ13C was evaluated with a generalized linear model with a Gaussian distribution due to non‐normality. NS denotes no significance, * indicates significant at p < 0.05; ** indicates significance at p < 0.01, and *** indicate significance at p < 0.001. n = 4, Land use d.f. = 2; Depth d.f. = 3; Season d.f. = 1; Land use*Depth d.f. = 6; Land use*Season d.f. = 2; Depth*Season d.f. = 3; Land use*Depth*Season d.f. = 6. Table S2: Mean ± standard error of soil moisture content (%) for forest, pasture, and cropland sites at 0–10, 30–40, 60–70, and 85–100 cm depths in spring and fall. Different uppercase letters indicate significant pairwise differences within one depth and season among land uses (p < 0.05); n = 4, d.f. = 2. Different lowercase letters indicate significant pairwise differences within one land use and season among depths (p < 0.05); n = 4, d.f. = 3. Asterisks indicate significant difference between seasons for the same land use and depth (p < 0.05); n = 4, d.f. = 3. Table S3: [file GCB-32-e70860-s001.docx]

# **Title: Seasonal flooding disrupts expected depth-dependent patterns of mineral associated carbon, nitrogen, and phosphorus across land uses**

**Running Title:** MAOM C, N, and P profile in saturated soils

Hannah P. Lieberman^*1^, Christian von Sperber^2^, Rachael Harman-Denhoed^1^, A. Stuart Grandy^3^, Cynthia M. Kallenbach^1,4^

^1^ Department of Natural Resource Sciences, Macdonald Campus of McGill University, Sainte-Anne-de-Bellevue, Québec, H9X 3V9, Canada

^2^ Department of Geography, McGill University, Montréal, Québec, H3A 0B9, Canada

^3^Department of Natural Resources and the Environment, University of New Hampshire, Durham, New Hampshire, 03824, U.S.

^4^Faculty of Land and Food Systems, University of British Columbia, Vancouver, British Columbia, V6T1Z4, Canada

* Author for correspondence: Hannah Lieberman; [hannah.lieberman@mail.mcgill.ca](mailto:hannah.lieberman@mail.mcgill.ca)

Keywords: organic matter persistence, mineral associated organic matter, seasonal flooding, subsoil, carbon, nitrogen, phosphorus, land use

**Supplemental information**

**
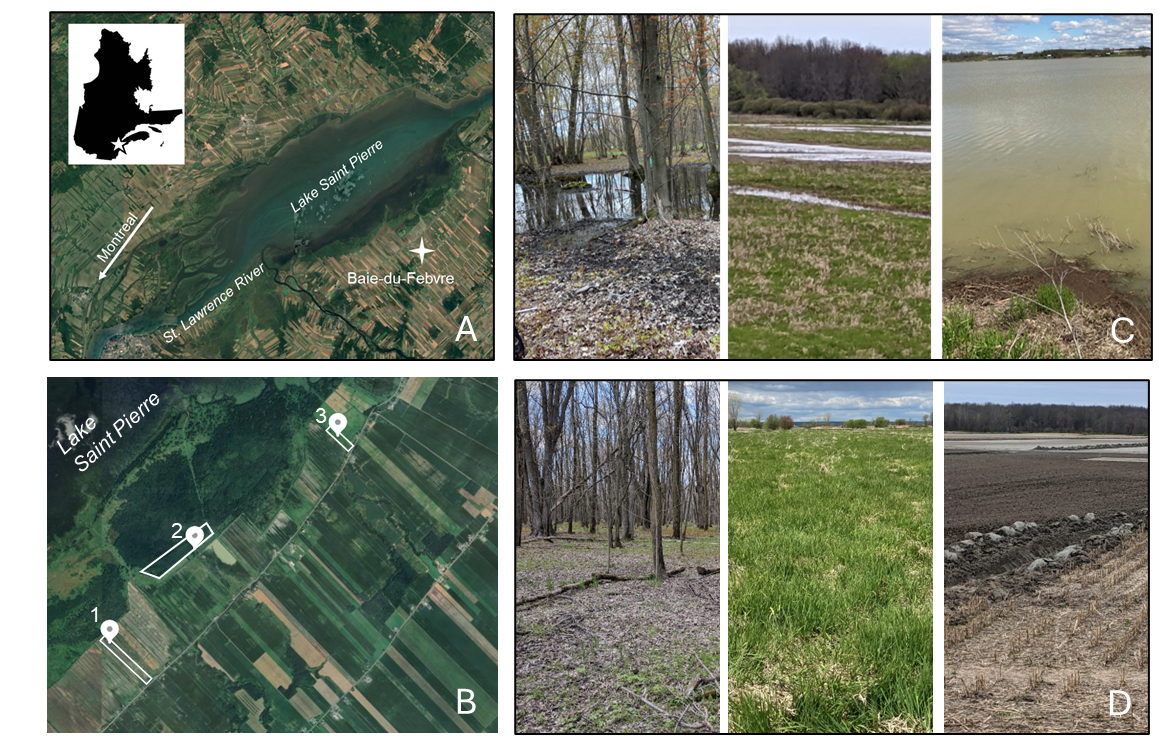
**

Supplemental Figure 1. Location of sampling sites and the land use gradient. Soil samples were collected near Baie-Du-Febvre, Quebec Canada (A) and included 3 different land uses (B): a cropland with a corn and soybean rotation under standard practices of the region (1); a native Maple-dominated forest (2); and a managed pasture (3). Land use sites experience near-annual spring flooding from Lake Saint Pierre and river tributaries (C), and May 2021 samples were collected 2 weeks after flood retreat (D). Images of land uses are forest, pasture, and cropland, left to right.

Supplemental Methods 1. Stable isotope calculations.

We calibrated the δX values against international reference materials from the United States Geological Survey (USGS) and International Atomic Energy Agency (IAEA; USGS40, USGS41a, IAEA-612, IAEA-N-2). The isotope ratio was calculated for δX as:

$$\text{δX}\text{ = }\frac{\text{Rsample}\text{ - Rreference}}{\text{Rreference}}\text{ x 1000}$$

As our N concentrations were below detection limits of the instrument, we used a N spike followed by a mixing model to calculate the final ^15^N values.

Supplemental Methods 2. Carbon use efficiency methodological details.

Before the carbon use efficiency (CUE) assay, 20 g dry weight equivalent of slowly thawed soils were pre-incubated for 24 hours. We then added 50 mg C g^1^ dry soil of 25 atom% labeled ^13^C glutamic acid (<1% total soil C) to each soil and incubated these at 20 °C for 16 hours. Both the glutamic acid label and 16-hour incubation period were selected based on preliminary respiration curves that captured an incubation time and substrate in which each treatment used most of the substrate but before substrate recycling, without biasing a depth or land use. After the incubation, a 20 ml CO_2_ sample was collected in an evacuated 12 mL Exetainer vial and MB^13^C was extracted using the MBC protocol described in section 2.7. These extractions were then freeze-dried and analyzed for ^13^C using Thermo Delta V IRMS interfaced to a NC2500 elemental analyzer at the Cornell University Stable Isotope Laboratory (COIL; Ithaca, NY, USA). Gas samples were analyzed on a Thermo Delta V IRMS interfaced to a Gas Bench II at the same facility.

CUE was calculated as:

$$\text{CUE = }\frac{\text{M}\text{B}^{\text{13}}\text{C}}{\text{(M}\text{B}^{\text{13}}\text{C}\text{+}^{\text{13}}\text{CO}_{\text{2}}\text{-C)}}\text{ x 100}$$

MB^13^C is the amount of substrate C incorporated into MBC and ^13^CO_2_-C is the substrate C respired as CO_2_.

MB^13^C and ^13^CO_2_-C was calculated as:

$$\text{MB}\text{13}\text{C or }\text{13}\text{CO}_{\text{2}}\text{-C =} \frac{\text{μg C g }\text{soil}^{\text{-1}}\text{ *(δcontrol - δsample)}}{\text{(δcontrol - δlabel)}}$$

Where μg C g soil^-1^ is either total MBC or total respired CO_2_, δcontrol is the δ^13^C value of MBC or CO_2_-C of the control-samples with unlabeled glutamic acid added, δsample is the δ^13^C value of MBC or CO_2_-C of labeled soil, and δlabel is the δ^13^C of the labeled substrate.

Supplemental Table 1. Three-way ANOVA results for land use by depth by season for soil moisture content (SMC; %), mineral associated (MAOM; g kg^-1^ dry soil) pools, MAOM δ^13^C and δ^15^N (‰), water extracted (WE; mg kg^-1^ dry soil) pools, microbial biomass C and N (MBC, MBN; mg kg^-1^ dry soil), and carbon use efficiency (CUE; %). Mineral associated δ^13^C, was evaluated with a generalized linear model with a Gaussian distribution due to non-normality. NS denotes no significance, * indicates significant at p<0.05; ** indicates significance at p<0.01, and *** indicate significance at p<0.001. *n=4, Land use d.f. = 2; Depth d.f. = 3; Season d.f. = 1; Land use*Depth d.f. =6; Land use*Season d.f. = 2; Depth*Season d.f. = 3; Land use*Depth*Season d.f. = 6.*

| Variable | Land use | Depth | Season | Land use*Depth | Land use*Season | Depth*Season | Land use*Depth*Season |
| --- | --- | --- | --- | --- | --- | --- | --- |
| SMC (%) | *** | *** | *** | *** | *** | *** | *** |
| MAOM C | *** | *** | NS | *** | NS | NS | NS |
| MAOM N | *** | *** | *** | *** | NS | NS | * |
| MAOM P | *** | *** | NS | *** | NS | * | ** |
| MAOM C:N | *** | *** | *** | *** | NS | * | NS |
| MAOM C:P | *** | *** | NS | *** | NS | NS | NS |
| MAOM N:P | *** | *** | *** | *** | * | NS | * |
| MAOM Fe | *** | *** | NS | *** | NS | NS | NS |
| MAOM Al | NS | *** | NS | *** | NS | NS | NS |
| MAOM Ca | *** | *** | NS | *** | NS | NS | NS |
| MAOM δ^13^C | NS | *** | NS | ** | NS | NS | NS |
| MAOM δ^15^N | ** | *** | NS | * | NS | NS | NS |
| WEOC | *** | *** | NS | *** | NS | * | NS |
| WEON | *** | *** | NS | *** | NS | NS | NS |
| WEP | *** | *** | ** | *** | ** | NS | NS |
| WEOM C:N | *** | *** | ** | ** | NS | NS | NS |
| WEOM C:P | *** | *** | NS | *** | NS | NS | NS |
| WEOM N:P | *** | *** | ** | *** | NS | NS | NS |
| MBC | NS | *** | *** | *** | NS | *** | NS |
| MBN | *** | *** | * | *** | NS | NS | NS |
| CUE | ** | *** | NS | NS | NS | NS | NS |

Supplemental Table 2. Mean ± standard error of soil moisture content (%) for forest, pasture, and cropland sites at 0-10, 30-40, 60-70 and 85-100 cm depths in spring and fall. Different upper-case letters indicate significant pairwise differences within one depth and season among land uses (p<0.05); *n*=4, *d.f. =* 2. Different lower-case letters indicate significant pairwise differences within one land-use and season among depths (p<0.05); *n*=4, *d.f. =* 3. Asterisks indicate significant difference between seasons for the same land use and depth (p<0.05); *n*=4, *d.f. =* 3.

| Land use | Depth (cm) | Spring soil moisture content (%) | Fall soil moisture content (%) |
| --- | --- | --- | --- |
| Forest | 0-10 | 101.8±12.7^Aa*^ | 35.7±2.0^Aa^ |
|  | 30-40 | 35.4±1.8^Ab*^ | 25.8±1.1^Ab^ |
|  | 60-70 | 39.1±1.9^Ab*^ | 26.7±1.1^Aab^ |
|  | 85-100 | 42.0±1.9^Ab*^ | 32.7±2.2^Aab^ |
| Pasture | 0-10 | 36.0±0.5^Ba^ | 31.5±0.6^Aa^ |
|  | 30-40 | 33.7±2.8^Aa^ | 33.1±1.4^Aa^ |
|  | 60-70 | 39.3±1.3^Aa^ | 34.6±1.2^Aa^ |
|  | 85-100 | 47.0±2.4^Ab^ | 48.5±0.6^Bb^ |
| Cropland | 0-10 | 25.5±1.2^Ca^ | 21.7±0.5^Ba^ |
|  | 30-40 | 28.2±2.8^Aa^ | 24.2±0.8^Aa^ |
|  | 60-70 | 39.6±3.2^Ab^ | 34.3±1.4^Ab^ |
|  | 85-100 | 41.7±1.3^Ab^ | 40.3±1.8^Bb^ |

Supplemental Table 3. Mean ± standard error of mineral associated N:P, and natural abundance isotope ratio of mineral associated δ^15^N (‰) for forest, pasture, and cropland sites at 0-10, 30-40, 60-70 and 85-100 cm depths with seasons combined. Different upper-case letters indicate significant pairwise differences within one depth between land uses (p<0.05); *n*=8, *d.f.* 2. Different lower-case letters indicate significant pairwise differences within one land use between depths (p<0.05); *n*=8, *d.f. =* 3.

| Land use | Depth (cm) | MAOM N:P | MAOM δ^15^N (‰) |
| --- | --- | --- | --- |
| Forest | 0-10 | 6.1±0.8^Aa^ | 2.5±0.2^Aa^ |
|  | 30-40 | 0.8±0.1^Bb^ | 3.1±0.3^Aa^ |
|  | 60-70 | 0.8±0.1^Ab^ | 3.4±0.3^Aa^ |
|  | 85-100 | 0.6±0.1^Ab^ | 2.0±0.5^Aa^ |
| Pasture | 0-10 | 1.9±0.2^Ba^ | 4.8±0.1^Ba^ |
|  | 30-40 | 2.7±0.3^Aa^ | 2.9±0.2^Aab^ |
|  | 60-70 | 0.5±0.1^Ab^ | 4.0±0.5^Aa^ |
|  | 85-100 | 0.5±01^Ab^ | 2.7±0.5^Ab^ |
| Cropland | 0-10 | 2.7±0.4^Ba^ | 4.3±0.3^ABab^ |
|  | 30-40 | 0.6±0.1^Bb^ | 3.0±0.5^Aab^ |
|  | 60-70 | 0.7±0.0^Ab^ | 4.5±0.4^Ab^ |
|  | 85-100 | 0.5±0.1^Ab^ | 2.5±0.8^Aa^ |

Supplemental Table 4. Two-way ANOVA results for land use by depth for the relative abundance of each compound class (%) of mineral associated compounds and plant litter for forest, pasture, and cropland sites at 0-10, 30-40, 60-70 and 85-100 cm depths in the spring. NS denotes no significance, * indicates significant at p<0.05, ** indicates significance at p<0.01, and *** indicate significance at p<0.001. *n=4, Land use d.f. = 2; Depth d.f. = 4; Land use*Depth d.f.=8.*

| Class | Land use | Depth | Land use*Depth |
| --- | --- | --- | --- |
| Aromatic | NS | ** | NS |
| Lignin | ** | *** | *** |
| Lipid | NS | *** | NS |
| N Bearing | *** | *** | NS |
| Phenolics | * | *** | NS |
| Polysaccharide | *** | *** | ** |
| Protein | NS | NS | NS |
| Unknown Origin | ** | *** | NS |

Supplemental Table 5. Two-way ANOVA results for land use by depth for the relative abundance (%) of mineral-associated compounds by log K_ow_ level for forest, pasture, cropland at 0-10, 30-40, 60-70 and 85-100 cm depths during the spring. NS denotes no significance, * indicates significant at p<0.05, ** indicates significance at p<0.01, and *** indicate significance at p<0.001. *n=4, Land use d.f. = 2; Depth d.f. = 3; Land use*Depth d.f.=6.*

| Log K_ow_ level | Land use | Depth | Land use*Depth |
| --- | --- | --- | --- |
| Soluble (<1) | *** | *** | ** |
| Slightly Insoluble (1-2) | *** | *** | ** |
| Insoluble (2-5) | NS | *** | NS |
| Highly Insoluble (>5) | NS | ** | NS |

Supplemental Table 6. Mean ± standard error of water extracted organic C (WEOC; mg kg^-1^ dry soil), water extracted N (WEN; mg kg^-1^ dry soil), water extracted inorganic P (WEP; mg kg^-1^ dry soil), water extract C:N (WE-C:N), water extract C:P (WE-C:P), and water extract N:P (WE-N:P) for forest, pasture, and cropland sites at 0-10, 30-40, 60-70 and 85-100 cm depths with seasons combined. Different upper-case letters indicate significant pairwise differences within one depth among land uses (p<0.05); *n*=8, *d.f. =* 2. Different lower-case letters indicate significant pairwise differences within one land use among depths (p<0.05); *n*=8, *d.f. =* 3.

| Land use | Depth | WEOC | WEN | WEP | WE-C:N | WE-C:P | WE-N:P |
| --- | --- | --- | --- | --- | --- | --- | --- |
| Forest | 0-10 | 203.0±16.5^Aa^ | 35.1±6.4^Aa^ | 1.4±0.4^Aa^ | 7.2±0.8^Aa^ | 226.6±50.5^Aa^ | 30.0±4.3^Aa^ |
|  | 30-40 | 41.7±5.3^Ab^ | 2.1±0.2^ABb^ | 2.7±0.7^Aa^ | 20.0±2.0^Aa^ | 31.8±10.8^Ab^ | 1.9±0.8^Ab^ |
|  | 60-70 | 22.5±4.1^Ab^ | 2.0±0.7^Ab^ | 0.5±0.1^Bb^ | 20.4±5.3^Aa^ | 80.5±37.7^Aa^ | 7.0±4.5^Abc^ |
|  | 85-100 | 20.49±2.8^Ab^ | 1.4±0.2^Ab^ | 0.3±0.1^Bb^ | 15.2±1.7^ABa^ | 99.1±21.3^Aa^ | 6.7±1.2^Ac^ |
| Pasture | 0-10 | 71.7±13.8^Ba^ | 7.3±0.6^Ba^ | 1.2±0.1^Aa^ | 9.6±1.7^Aa^ | 67.3±19.2^ABa^ | 6.2±0.8^Ba^ |
|  | 30-40 | 55.0±12.9^Aa^ | 6.3±1.7^Aa^ | 1.0±0.1^Aa^ | 13.7±4.5^Aa^ | 60.1±18.8^Aa^ | 8.2±2.4^Ba^ |
|  | 60-70 | 23.0±4.5^Ab^ | 1.3±0.3^Ab^ | 3.4±0.5^Aa^ | 31.5±11.8^Aab^ | 7.4±1.2^Bb^ | 0.4±0.1^Bb^ |
|  | 85-100 | 40.5±10.3^Aa^ | 1.0±0.2^Ab^ | 1.1±0.2^Aa^ | 46.3±12.5^Ab^ | 41.6±13.3^Aab^ | 1.1±0.3^Bb^ |
| Cropland | 0-10 | 32.1±12.2^Ba^ | 6.7±1.4^Ba^ | 1.4±0.2^Aa^ | 5.3±2.5^Aa^ | 28.1±14.8^Ba^ | 4.0±0.9^Bab^ |
|  | 30-40 | 14.9±1.6^Ba^ | 1.6±0.4^Bb^ | 1.0±0.2^Aa^ | 12.2±2.8^Ab^ | 65.3±43.2^Aab^ | 6.2±4.3^ABa^ |
|  | 60-70 | 6.5±1.3^Bb^ | 0.8± 0.2^Ab^ | 0.2±0.1^Bb^ | 10.3±3.0^Aab^ | 37.9±9.5^ABab^ | 4.7±1.4^Aa^ |
|  | 85-100 | 8.6±2.2^Bb^ | 1.4±0.2^Ab^ | 0.1±0.0^Bb^ | 6.7±1.7^Bab^ | 196.6±122.4^Ab^ | 23.4±7.9^Ab^ |

Supplemental Table 7. Mean ± standard error of carbon use efficiency (%) for forest, pasture, and cropland sites at 0-10 and 85-100 cm depths with seasons combined. Different uppercase letters indicate significant pairwise differences within one depth among land uses (p<0.05); *n*=8, *d.f. =* 2. Different lower-case letters indicate significant pairwise differences within one land use among depths (p<0.05); *n*=8, *d.f. =* 3.

| Land use | Depth (cm) | Carbon use efficiency (%) |
| --- | --- | --- |
| Forest | 0-10 | 43.40±3.7 ^Ba^ |
|  | 85-100 | 93.63±3.4^Ab^ |
| Pasture | 0-10 | 54.19±3.8 ^ABa^ |
|  | 85-100 | 92.48±1.1^Ab^ |
| Cropland | 0-10 | 61.94±3.2 ^Aa^ |
|  | 85-100 | 95.91±1.1^Ab^ |

| Land use | Depth (cm) | MBC  (mg kg^-1^) | MBN  (mg kg^-1^) |
| --- | --- | --- | --- |
| Forest | 0-10 | 2060.5±369.6^Aa^ | 235.8±39.5^Aa^ |
|  | 30-40 | 205.6±66.1^Ab^ | 6.2±1.9^Ab^ |
|  | 60-70 | 282.1±89.9^Ab^ | 5.1±1.2^Ab^ |
|  | 85-100 | 377.8±62.7^Ab^ | 7.3±1.9^Ab^ |
| Pasture | 0-10 | 597.7±85.0^Ba^ | 95.8±17.0^ABa^ |
|  | 30-40 | 291.7±122.9^Aa^ | 34.3±4.6^Ba^ |
|  | 60-70 | 268.4±68.3^Aa^ | 8.6±3.4^Ab^ |
|  | 85-100 | 387.4±48.2^Aa^ | 8.9±2.2^Ab^ |
| Cropland | 0-10 | 344.7±77.8^Ba^ | 38.3±4.9^Ba^ |
|  | 30-40 | 202.8±84.7^Aa^ | 3.3±0.5^Ab^ |
|  | 60-70 | 234.9±90.5^Aa^ | 4.0±1.0^Ab^ |
|  | 85-100 | 373.3±53.2^Aa^ | 9.5±2.4^Ab^ |

Supplemental Table 8. Mean ± standard error microbial biomass C (MBC; mg kg^-1^ dry soil) and microbial biomass N (MBN; mg kg^-1^ dry soil) for forest, pasture, and cropland sites at 0-10, 30-40, 60-70 and 85-100 cm depths with seasons combined. Due to limited sample, we were unable to conduct these analyses on MBC in the Forest at 0-10cm for the fall. Different uppercase letters indicate significant pairwise differences within one depth among land uses (p<0.05); *n*=8, *d.f. =* 2. Different lower-case letters indicate significant pairwise differences within one land use among depths (p<0.05); *n*=8, *d.f. =* 3.
